# Bioactive Inorganic Materials for Innervated Multi‐Tissue Regeneration

**DOI:** 10.1002/advs.202415344

**Published:** 2025-02-27

**Authors:** Hongjian Zhang, Ziyi Zhao, Chengtie Wu

**Affiliations:** ^1^ State Key Laboratory of High Performance Ceramics and Superfine Microstructure Shanghai Institute of Ceramics Chinese Academy of Sciences Shanghai 200050 P. R. China; ^2^ Center of Materials Science and Optoelectronics Engineering University of Chinese Academy of Sciences Beijing 100049 P. R. China

**Keywords:** functional recovery, inorganic biomaterials, innervated tissue regeneration, tissue engineering and regenerative medicine

## Abstract

Tissue engineering aims to repair damaged tissues with physiological functions recovery. Although several therapeutic strategies are there for tissue regeneration, the functional recovery of regenerated tissues still poses significant challenges due to the lack of concerns of tissue innervation. Design rationale of multifunctional biomaterials with both tissue‐induction and neural induction activities shows great potential for functional tissue regeneration. Recently, the research and application of inorganic biomaterials attracts increasing attention in innervated multi‐tissue regeneration, such as central nerves, bone, and skin, because of its superior tunable chemical composition, topographical structures, and physiochemical properties. More importantly, inorganic biomaterials are easily combined with other organic materials, biological factors, and external stimuli to enhance their therapeutic effects. This review presents a comprehensive overview of recent advancements of inorganic biomaterials for innervated multi‐tissue regeneration. It begins with introducing classification and properties of typical inorganic biomaterials and design rationale of inorganic‐based material composites. Then, recent progresses of inorganic biomaterials in regenerating various nerves and nerve‐innervated tissues with functional recovery are systematically reviewed. Finally, the existing challenges and future perspectives are proposed. This review may pave the way for the direction of inorganic biomaterials and offers a new strategy for tissue regeneration in combination of innervation.

## Graphical abstract

This review comprehensively summarizes the recent advancements of inorganic biomaterials for innervated multi‐tissue regeneration. It emphasizes the design principles of inorganic‐based material composites and their application in functional regeneration of nerves, bone, muscles, skin, and cavernous tissues. It is anticipated that this review may provide a new sight for designing novel biomaterials for functional tissue regeneration and clinical translation.

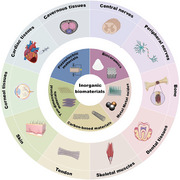

## 1 Introduction

The regeneration and functional recovery of human tissue/organs defects caused by accidental trauma, surgery, and diseases remain challenging in clinical settings. Tissue engineering has emerged as promising approach to regenerate or replace injured tissues/organs, which is regarded as an alternative of traditional autografts and allografts that have the risk of donor shortage, immune rejection, and diseases transmission.^[^ ^1^ ^]^ Currently, biomaterials with tunable composition, structures, and physicochemical properties could mimic the extracellular microenvironments to support cell adhesion, proliferation, and specific differentiation, thereby inducing tissue regeneration. However, the restoration of physiological functions of regenerated tissues is still in its early stages due to key factors of the nerves innervation of biomaterials having been largely overlooked.^[^ ^2^ ^]^ The nervous system composed of central nervous system and peripheral nervous system is the most complex and important part of the human body, among which central nerves containing the brain and spinal cord serve as commander to integrate sensory and motor functions; while, peripheral nerves are responsible for receiving, processing, and transmitting signals between the central nervous system and targeted tissues/organs.^[^ ^3^ ^]^ It should be noted that peripheral nerves are densely distributed in many tissues (e.g., bone, skin, muscles, and heart) and regulate their development, regeneration, and physiological functions.^[^ ^2^ ^]^ Nerves could regulate tissue‐resident cells behaviors and immune microenvironments via secreting numerous neuropeptides and neurotrophic factors.^[^ ^4^ ^]^ Series studies have found that denervation, usually accompanied with the outcomes of delayed healing and poor tissue integration, further demonstrated the guidance role of nerves.^[^ ^5^ ^]^ In clinical settings, autologous transplantation showed satisfactory therapeutic effects because they could preserve nerve fibers and enable timely integration with host systems.^[^ ^6^ ^]^ More importantly, the physiological functions of tissues such as contraction and perception were mediated by innervated nerves. Hence, rapid integration with host nervous systems could not only accelerate healing efficiency but also help to recover the physiological functions of regenerated tissues.^[^ ^7^ ^]^ Both are beneficial to improve the life quality of patients. Therefore, it is of great significance to develop novel tissue engineering scaffolds with neuroregulatory properties, which have the potential to achieve functional tissue regeneration.

In all, the above scaffolds should simultaneously possess both tissue‐induction activity and neural induction activity. Specifically, tissue‐induction means the capacity of regulating the adhesion, proliferation, and differentiation of tissue‐resident cells. For example, bone regenerative scaffolds need to promote the osteogenic differentiation of bone mesenchymal stem cells (BMSCs) and induce mineral deposition. Skin tissue engineering scaffolds should have the ability of promoting re‐epithelialization and collagen deposition. On the other hand, neural activity represents the ability of biomaterials that induce neurite outgrowth and regeneration, myelination, and neuronal differentiation of stem cells; these contribute to tissue innervation. Moreover, the close interaction of nerves and tissue‐resident cells should be fully considered. For example, nerves and blood vessels within tissues are distributed in parallel anatomically and share some common signals to promote the development of each other.^[^ ^8^ ^]^ Neuropeptides secreted from nerves may promote angiogenesis; while, blood vessels provide oxygen and nutrients to support neurons activity.^[^ ^9^ ^]^ Besides, nerves‐derived cytokines have the ability of regulating macrophages polarized from M1 toward M2 phenotype to create a pro‐regenerative microenvironment.^[^ ^10^ ^]^ However, neural activity is often ignored when designing tissue regenerative scaffolds.

Recently, owing to its favorable biocompatibility, biodegradability, and adjustable physicochemical properties, a series of inorganic biomaterials‐based tissue engineering scaffolds exhibited both tissue‐induction activity and neural induction activity that had been explored for innervated tissue regeneration. Specifically, multiple bioactive ions released from inorganic biomaterials could create a beneficial ionic microenvironment to regulate cell behaviors and enhance neural activity. Several ions including Ca and Mg ions have been confirmed to be beneficial to tissue regeneration and neurogenesis.^[^ ^11^ ^]^ Besides, inorganic biomaterials are easily modified with micro/nano topographical structures, which provide physical cues for inducing cell differentiation and neurites outgrowth. Moreover, electroactive inorganic biomaterials could directly transmit electric signals or generate electrical currents under external stimulations to mimic the bioelectricity of natural tissues/organs.^[^ ^12^ ^]^ Overall, inorganic biomaterials could provide chemical, physical, and electrical cues to inducing tissues regeneration and innervation through releasing bioactive ions, creating micro/nano topographical structures, and transmitting/generating electric signals. This is despite the fact that during the past few decades, inorganic biomaterials have been extensively explored and systematically summarized in anti‐bacterial, hemostasis, and cancer theranostics.^[^ ^13^ ^]^ However, there are few reviews that focus on the application of inorganic biomaterials in the fields of nerves and nerve‐innervated tissues regeneration. With the increasing attention of inorganic biomaterials in functional tissue regeneration, it is timely and necessary to systematically summarize the recent progress of inorganic‐based biomaterial composites and their application in nerves and nerve‐innervated tissues regeneration.

**Figure 1:**
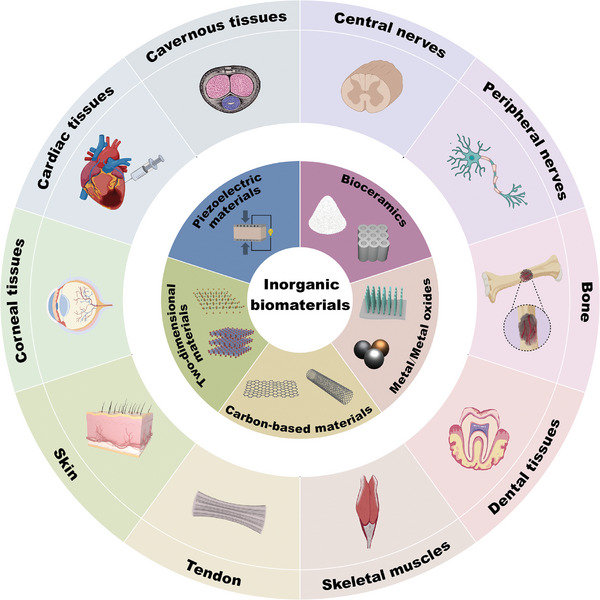
Schematic illustration of the recent advancements of inorganic biomaterials for innervated tissue regeneration.

In this review, we aim to overview the recent advancements of inorganic biomaterials for nerves and nerve‐innervated tissues regeneration (**Figure** **1**). First, we introduce the types and properties of the most studied inorganic biomaterials, including bioceramics, metal/metal oxides, carbon‐based materials, 2D materials, and piezoelectric materials. Subsequently, four types of inorganic‐based material composites used for innervated tissue regeneration are described. Then, the application of inorganic‐based materials in promoting the regeneration of various nerves and nerve‐innervated tissues including central nerves, peripheral nerves, bone, dental tissue, skeletal muscle, tendon, skin, corneal tissues, cardiac tissues, and cavernous tissues will be presented. Finally, the prospects and future research directions of inorganic biomaterials on functional tissues regeneration are discussed. This review highlights the great significance of inorganic biomaterials in innervated multi‐tissue regeneration, which may provide a new point of view for designing tissue engineering scaffolds for functional tissue regeneration.

## 2 Classification and Properties of Typical Inorganic Biomaterials

**Figure 2:**
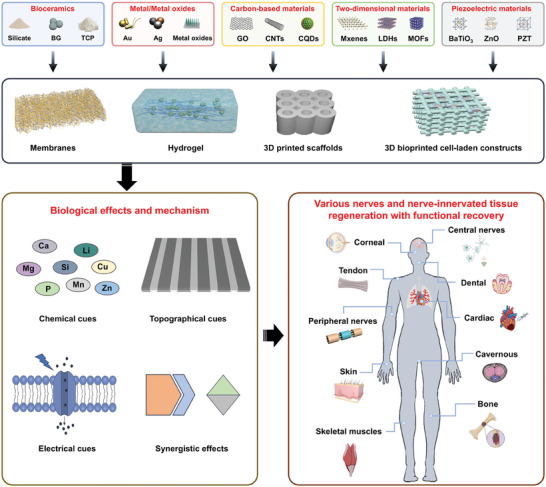
A summary and outline of typical inorganic biomaterials and inorganic‐based material composites, their biological effects, and application in innervated multi‐tissue regeneration.

During the past few decades, a series of inorganic biomaterials has been developed for tissue engineering and regenerative medicine. In this section, we mainly introduce the typical inorganic biomaterials that have been confirmed to possess excellent both neural‐induction activity and tissue induction activity, such as bioceramics, metal/metal oxides, carbon‐based materials, 2D materials, and piezoelectric materials (**Figure** **2**). The synthetic strategies of inorganic biomaterials including top–down (e.g., mechanical exfoliation and liquid phase ultrasonic exfoliation) and bottom–up (e.g., hydrothermal, solvothermal, sol–gel) approaches are briefly introduced, and their properties and bioactivity are also mentioned.

### 2.1 Bioceramics

Bioceramics are a type of ceramics materials that has been widely used for the repair and regeneration of bone and dental tissues for several years.^[^ ^11^ ^]^ Broadly speaking, bioceramics contain both crystalline and amorphous inorganic biomaterials, including silicate‐based ceramics, phosphate‐based ceramics, bioactive glasses, and their composites. In 1969, Prof. Larry Hench found that bioglass composed of 45% SiO_2_, 24.5% Na_2_O, 24.5% CaO, and 6% P_2_O_5_ could react with host bone tissues to form tight interfacial bonding after implantation in vivo, opening the curtain of bioactive ceramics research.^[^ ^14^ ^]^ During the past few decades, the application field of bioceramics has been expanded from simple hard tissue regeneration to complex soft tissue regeneration (e.g., skin, skeletal muscle, cartilage, tendon, heart, and brain), anti‐bacterial, tumor therapy, and diagnosis.^[^ ^15^ ^]^ At present, a series of methods including solid‐phase reaction, sol–gel, hydrothermal, solvothermal, co‐precipitation, and containerless melting has been developed to fabricate bioceramics. Their chemical composition, size, shapes, surface charge, and ionic release profile can be precisely controlled via changing reaction parameters such as reactant concentration, temperature, time, and pH. Bioceramics have good biocompatibility and degradability, which enables release of multiple bioactive ions to create beneficial ionic microenvironments for regulating cell behaviors including adhesion, proliferation, and specific differentiation. For example, Ca is the main component of bone and teeth, which enables promotion of the osteogenic differentiation of MSCs and mineral deposition.^[^ ^16^ ^]^ Ca can react with phosphate ions to induce the formation of hydroxyapatite. As the second messenger, Ca is widely involved in signal transduction of various biological processes and plays crucial roles in neurogenesis.^[^ ^17^ ^]^ Mg is the essential element of human body, which plays great important roles in cell metabolism and tissue/organ development. It is reported that Mg can promote the proliferation and differentiation of osteoblast through melastatin transient receptor potential channel 7 (TRPM7) and magnesium transporter 1 (MagT1)‐mediated signal pathway.^[^ ^18^ ^]^ Besides, Mg possesses neuroactivity that enables inducing neurons maturation and secreting neuropeptides calcitonin gene‐related peptide (CGRP), which further enhance bone regeneration.^[^ ^19^ ^]^ In addition, it is known that inflammation is a natural process by which M1‐macrophages (pro‐inflammatory phenotype) are responsible for clearing microorganisms and cellular debris; while, M2‐macrophages (anti‐inflammatory phenotype) can secrete regenerative factors for angiogenesis and tissue repair.^[^ ^20^ ^]^ Hence, timely and sequential polarization of macrophage from M1 (pro‐inflammatory) to M2 (anti‐inflammatory) phenotype could help to achieve a more complete healing process after injury.^[^ ^21^ ^]^ It is reported that Mg ions could timely and sequentially induce the polarization of macrophages from M1 toward M2 phenotype to build pro‐regenerative microenvironments.^[^ ^22^ ^]^ During the past two decades, our groups have systematically explored the multifunctional bioactivity of Si ions, including osteogenesis, angiogenesis, neurogenesis, immunomodulation, and cell–cell interactions. For example, Si ions could promote the osteogenic differentiation through activating AMPK/ERK 1/2 and PI3K/AKT pathway.^[^ ^23^ ^]^ Moreover, Si could also induce the axon growth and enhance the secretion of semaphorin 3A (Sema3A) of neurons, which further promote osteogenesis and angiogenesis activities.^[^ ^24^ ^]^ In addition to the above‐mentioned, multiple bioactive ions (e.g., Sr, Cu, Mn, Zn, and Mo) have been confirmed to possess superior bioactivities that actively participate in cell signaling and differentiation.^[^ ^25^ ^]^ More interestingly, different types of ions exhibited synergistic effects on regulating cell behaviors including proliferation and genes expression. For example, as compared with single Cu or Si ions, the combination of Cu and Si ions showed remarkable promotion effects on the angiogenic‐related genes expression of endothelial cells.^[^ ^26^ ^]^ Another study found that Li, Mg, and Si ions could synergistically promote osteogenic differentiation of BMSCs and chondrocyte maturation.^[^ ^27^ ^]^ Therefore, the combination of multiple bioactive ions represents an effective and promising approach for regulating cell behaviors.

Apart from releasing bioactive ions, the structural topographical cues of bioceramics should also not be ignored because of its crucial effects on regulating cell fate.^[^ ^28^ ^]^ In recent years, researchers have developed various methods (e.g., templating, hydrothermal, bidirectional freezing, and 3D printing) to endow bioceramics with controllable micro/nano topographical structures and further investigate their effects on cell differentiation.^[^ ^29^ ^]^ For example, Deng et al. had combined 3D printing and hydrothermal methods to prepare surface micro/nano structural bioceramic scaffolds for osteochondral regeneration.^[^ ^30^ ^]^ 3D printed bredigite bioceramic scaffolds were treated with a hydrothermal process under different conditions (reactive concentrations and reaction time) to generate various morphology calcium phosphate crystals on the surface of filament. The micro/nano structures could activate integrin‐related proteins αvβ1 and α5β1 signals to promote chondrocytes maturation and activate the integrin α5β1 and RhoA pathway to induce osteogenesis. Another study reported that surface flower‐liker nanostructures‐modified calcium silicate bioceramics prepared via hydrothermal treatment exhibited better hydrophilicity and promoted the osteogenic differentiation of BMSCs via activating FAK/p38 signal pathway.^[^ ^31^ ^]^ In another example, Zhang et al. had successfully prepared tree‐like bioceramic scaffolds with gradient surface microstructures via digital light processing (DLP)‐based 3D printing technique.^[^ ^32^ ^]^ The results showed that their gradient structures could enhance the osteogenic differentiation activity of BMSCs and neurogenic differentiation activity of Schwann cells in a thickness‐dependent manner. Currently, the mechanism of surface micro/nano structures regulating cell differentiation is controversial. Several studies have found that surface micro/nano structures of bioceramic scaffolds could induce the selective adsorption of proteins such as fibronectin and vitronectin to form different cell niche microenvironments, which further affect the cell adhesion and differentiation behaviors.^[^ ^33^ ^]^ Generally, both chemical composition such as bioactive ions release and structural characteristics such as surface micro/nano structures play important roles in regulating cell behaviors and tissue regeneration. In fact, the chemical and structural characteristics of bioceramics usually exert their biological effects simultaneously after implantation in vivo. Hence, it is required to carefully design bioceramics with appropriate chemical and structural characteristics, which would help to clarify which feature is much more important for tissue regeneration.

### 2.2 Metal/Metal Oxides

Metal and metal oxides are one type of inorganic biomaterials that have been extensively applied in biomedical applications including drugs delivery, cancer therapy, tissue regeneration, and bioimaging due to its superior physiochemical properties such as high surface area, surface plasmon resonance, and high reactivity.^[^ ^13^, ^34^ ^]^ Commonly, metal/metal oxides can be synthesized by hydrothermal, solvothermal, and sol–gel methods with tunable size, morphology, and surface microstructures. Gold nanomaterials have the unique advantages of controllable shapes and sizes, biosafety, simple synthesis, and photothermal response, holding great potential in regenerative medicine.^[^ ^35^ ^]^ Previous studies have shown that Au nanoparticles promote the osteogenic differentiation of BMSCs via activating p38 MAPK signals and inducing angiogenesis.^[^ ^36^ ^]^ Au nanoparticles‐mediated mild photothermal effects also open the transient receptor potential vanilloid 1 (TRPV1) channel on the membranes of Schwann cells, further promoting neurotrophic factors (NGF and BDNF) expression and myelination.^[^ ^37^ ^]^ Besides, Au nanoparticles modified with biomacromolecules (e.g., DNA, mRNA, and proteins) can also enhance its targeting capability.^[^ ^38^ ^]^ Due to its inherent anti‐bacterial properties, Ag nanoparticles itself or combined with polymers were usually used for the treatment of infectious tissue injury including bone repair and wound healing.^[^ ^39^ ^]^ Ag ions had broad‐spectrum anti‐bacterial ability that destroyed the structures of bacterial cell walls and membranes, leading to the outflow of intra‐substances. Besides, low dose Ag ions can also promote the angiogenic‐related genes expression of endothelial cells; however, one needs to avoid high concentration of Ag ions that would damage DNA and induce cell apoptosis.^[^ ^40^ ^]^

Due to its high bioactivity and enzymes‐like properties, metal oxides possess the capacity of regulating cell metabolism, modulating inflammatory microenvironments, and generating therapeutic gas, which have been used in regenerative medicines.^[^ ^41^ ^]^ For instance, the excess generation and accumulation of ROS during the inflammatory process contribute to cells dysfunction and death, further impeding wound healing.^[^ ^42^ ^]^ Metal oxides including MnO_2_ and CeO_2_ nanoparticles have been confirmed to have excellent catalase‐like activity and are regarded as superior anti‐oxidants to scavenge reactive oxygen species (ROS) and catalyze the dismutation of H_2_O_2_ into H_2_O and O_2_; thus, alleviating inflammation of the injury area.^[^ ^43^ ^]^ Zhang et al. designed CeO_2_ nanoparticles‐incorporated 3D printed BG scaffolds to promote sequential bone regeneration. CeO_2_ nanoparticles can alleviate the early inflammatory response of injury, promote the osteogenic differentiation of BMSCs, and induce mineral deposition, which synergistically promotes bone regeneration.^[^ ^44^ ^]^ Moreover, iron oxides including Fe_3_O_4_ and γ‐Fe_2_O_3_ possess excellent superparamagnetic and biocompatibility properties, which hold potential in bioimaging and tissue engineering.^[^ ^45^ ^]^ Iron oxides have been approved by the Food and Drug Administration (FDA) for tracking the implanted exogenous cells in clinical settings. Besides, it is reported that Fe_3_O_4_ could induce the neurite outgrowth of neurons and myelination. Under magnetic fields, iron oxides could serve as massages to stimulate Schwann cells secreting large number of extracellular vesicles, which further promoted angiogenesis and nerves regeneration.^[^ ^46^ ^]^ In addition to single metal oxides, recent studies have confirmed that dual metal oxides exhibit enhanced catalytic activities and multiple enzymes‐like properties. For example, Cu–Fe_3_O_4_ nanocluster has triple enzyme activities (peroxidase, glutathione peroxidase, and superoxide dismutase), exhibiting excellent anti‐inflammatory and bactericidal effects, thereby effectively repairing infectious skin wounds.^[^ ^47^ ^]^

### 2.3 Carbon‐Based Materials

Due to its superior mechanical strength, chemical stability, high electrical conductivity, and biocompatibility, carbon‐based materials including graphene, graphene oxides (GO), reduced graphene oxides (rGO), single‐walled carbon nanotubes (SWCNTs), multi‐walled carbon nanotubes (MWCNTs), and carbon quantum dots (CQDs) have been extensively explored in biomedical applications varying from disease diagnosis and treatment to tissue regeneration during the past few decades.^[^ ^12^, ^48^ ^]^ Although the main component of carbon‐based materials is carbon elements, the carbon allotropic forms, size, and surface chemistry features endow them with different physiochemical properties. Generally, the synthesis of carbon‐based materials can be divided into top–down and bottom–up routes, among which top–down is widely used in the lab via mechanical exfoliation.^[^ ^49^ ^]^

Owing to its excellent physiochemical properties, various carbon‐based materials used alone or combined with external stimulation have been regarded as an effective approach to regulate stem cell behaviors such as proliferation and differentiation, especially in bone tissue engineering and neural tissue engineering.^[^ ^50^ ^]^ For example, GO was integrated with calcium phosphates to enhance its osteo‐conduction. The abundant functional groups of GO could adsorb cytokines and proteins, which further promoted mineral deposition and bone formation.^[^ ^48^ ^]^ Besides, adipose derived stem cells (ADSCs) cultured on the surface of graphene film could spontaneously differentiate into mature neurons under wireless electromagnetic fields. The mV‐level voltage generated from the graphene film could promote Ca^2+^ intracellular influx; thus, enhancing the neurogenic‐related genes and proteins expression.^[^ ^51^ ^]^ In addition, CNTs also exhibited suitable biological activity. It is reported that CNTs had good osteo‐induction and osteo‐conduction properties that promoted the osteogenic differentiation of BMSCs.^[^ ^52^ ^]^ Another study found that CNTs‐containing polyethylene glycol (PEG) hydrogel effectively promoted the long‐periods survival and differentiation of PC12 cells; while, also enhancing the neuronal networks activity, exhibiting great potential in nerve regeneration.^[^ ^53^ ^]^ Moreover, carbon‐based materials also affect the growth and development of organoids. It is reported that the addition of CNT into hydrogel matrixes can influence its viscoelasticity and subsequently activate Piezo and p38 MAPK‐YAP signal pathway to enhance intestinal organoids development. Besides, the internalization of CNTs also improves the mitochondrial respiration and nutrient absorption levels. These works inspired researchers to develop carbon materials‐based bone/neural organoids in the future.^[^ ^54^ ^]^

### 2.4 2D Materials

In recent years, various 2DMs including MXenes, phosphorus‐based nanomaterials, transition metal dichalcogenides (TMDCs), layered double hydroxides (LDHs), and metal–organic frameworks (MOFs) have been developed and applied for biomedicine.^[^ ^55^ ^]^ 2DMs have graphite‐like layered structures with the thickness less than 100 nm, mainly composed of single/few atomic layers.^[^ ^56^ ^]^ Generally, the preparation of 2DMs can be divided into top–down and bottom–up methods, among which top–down approaches were widely used in the lab, separating layered structural bulk materials via mechanical exfoliation and liquid phase ultrasonic exfoliation.^[^ ^57^ ^]^ Due to its special physiochemical properties including layered structures, large surface area, abundant functional groups, and high electrical conductivity, 2DMs have been used for tumor therapy, bioimaging, biosensors, and regenerative medicine.^[^ ^58^ ^]^ For example, the large surface area of 2DMs made it possible to serve as a delivery platform for loading and releasing bioactive agents including drugs, proteins, peptides, and genes.^[^ ^59^ ^]^ As compared with traditional oral administration or intravenous injection route, 2DMs‐loaded with bioactive agents could improve utilization efficiency via targeted delivery and protect bioactive agents from degradation.^[^ ^60^ ^]^ Moreover, the superior electroactive property of 2DMs could mimic the conductive microenvironment of native tissues; thus, facilitating the electrical signals transmission and regulating cell behaviors such as proliferation, adhesion, and differentiation.^[^ ^61^ ^]^

MXenes is a novel subcategory of 2D materials (2DMs), mainly including transition layered metal carbides, nitrides, and carbonitrides. The general formula of MXenes is M*_n_*_+1_X*_n_* T*_x_*, where *n* ranges from 1 to 3, M represents transition metals elements (e.g., Ti, Mo, and Ta), X represents C or N elements, T is the type of surface functional groups, usually ─OH, ─O, ─F, and ─Cl; while, *x* represents the number of functional groups.^[^ ^58b^ ^]^ Commonly, 2D layered MXenes were obtained by selectively etching parent ternary 3D MAX phases to remove the A‐groups elements. Owing to its large surface area, abundant functional groups, high electrical conductivity, and good biocompatibility, MXenes have been widely applied in biomedical applications.^[^ ^62^ ^]^ Besides, the large surface area and functional groups of MXenes make it possible of delivering drugs and growth factors to synergistically enhance the therapeutic effects.

Phosphorus‐based nanomaterials including black phosphorus (BP), germanium phosphide (GeP), and silicon phosphide (SiP) have attracted more attention in biomedical applications such as tissue regeneration, drug delivery, and theranostics.^[^ ^63^ ^]^ Phosphorus‐based nanomaterials possess many advantages in biomedical applications, mainly based on the following aspects: 1) phosphorus based‐biomaterials have suitable biodegradability in physiological environments and the main degradation product is phosphate ion, that is the crucial component of bone and tooth.^[^ ^64^ ^]^ 2) Phosphorus exists abundantly in nervous tissues and has remarkable promotion effects on neurogenesis via inducing axon extension and myelination.^[^ ^64b^ ^]^ 3) Phosphorus‐based nanomaterials have superior physicochemical properties including tunable bandgap, high surface area‐to‐volume ratio, and superior electrical conductivity, making them very sensitive to external stimulation such as electric and near infrared (NIR).^[^ ^59^ ^]^ Hence, phosphorus‐based smart responsive scaffolds are developed for precision medicine. Besides, their large surface area enables the loading and releasing of bioactive components, such as drugs, growth factors, and genes. Moreover, the electroactive property of phosphorus‐based nanomaterials can mimic the conductive microenvironment of native tissues; thus, facilitating the electrical signals transmission and regulating cell fates.^[^ ^65^ ^]^

However, phosphorus‐based nanomaterials also have some drawbacks such as poor physiological stability and being highly reactive to oxygen and water.^[^ ^66^ ^]^ It is reported that the metal cations modification via electrostatic and coordinate interactions can not only enhance the physiological stability of phosphorus‐based nanomaterials but also expand its biomedical applications.^[^ ^67^ ^]^ For instance, as compared with GeP nanosheets, GeP@Cu nanosheet exhibits superb anti‐bacterial, angiogenesis, neurogenesis capabilities, making them able to be used in infectious bone regeneration.^[^ ^68^ ^]^ Moreover, owing to the good promotion effects of Mg ions on neurogenesis and osteogenesis, the introduction of Mg ions into BP nanosheet also synergistically promotes the neurogenic differentiation of neural stem cells (NSCs) and osteogenic differentiation of BMSCs.^[^ ^67^ ^]^

TMDCs have sandwiched like‐layered structures composed of transition metal and chalcogen atoms layers, and these layers are bonded by weak van der Waals forces. The general formula of TMDCs is *MX*_2_, where *M* represents transition metal elements such as Ti, Nb, and Mo; while, *X* refers to chalcogens including S, Se, and Te. As compared with other 2DMs, TMDCs have the advantages of adjustable performance, rich raw materials, and easy preparation.^[^ ^69^ ^]^ Moreover, TMDCs possess good physiological stability and biocompatibility, enabling them to exerting long‐term therapeutic effects in organisms. It is noted that the long‐term biosafety needs to be fully considered, and its degradation product should also be biocompatible with the human body. Owing to these beneficial properties, TMDCs‐based biomaterials have been used in cancer therapy, tissue regeneration, drug delivery, and bioimaging.^[^ ^58a^ ^]^

LDH is another class of 2D nano‐clay materials with layered structures, which is composed of positively charged ionic layers and interlayer anions.^[^ ^70^ ^]^ LDH has tunable size and chemical composition (metal cations and interlayer anions types), as well as good ion‐exchange ability. Moreover, the large surface area, abundant functional groups, and multiple metal ions endow LDH with excellent bioactivities, making them extensively explored in biomedical applications.^[^ ^71^ ^]^ A series of LDH such as MgFe‐LDH, MgAl‐LDH, and MgMn‐LDH has been explored for tumor therapy, bone regeneration, and neural repair. For example, Mg‐containing LDH could stimulate osteogenesis and neurogenesis; thus, promoting innervated bone formation in vivo. Mn‐containing LDH enables to regulate the inflammatory microenvironments through scavenging ROS and generating O_2_, which could promote neural differentiation and neurite growth of PC12 cells.^[^ ^72^ ^]^ Moreover, it is very convenient to dope other nutritional elements such as Eu, Ce, and Gd to improve its biological performance and endow LDH with enhanced photothermal, anti‐bacterial, angiogenesis, and fluorescence detection properties.^[^ ^73^ ^]^ In addition, the large surface area and abundant functional groups of LDH enable to deliver growth factors without decreasing their bioactivity, thereby effectively promoting tissue regeneration.

MOFs is a kind of 2D porous material, composed of the self‐assembling of metal ions and organic linkers through coordination bonds.^[^ ^59^ ^]^ The size, morphology, and biological activity of MOFs can be easily modulated through selecting appropriate metal ions and organic linkers to meet the requirement of different biomedical applications. At present, a series of Zn‐, Cu‐, Fe‐, and Mg‐based MOFs has been explored for bone regeneration, wound healing, anti‐bacterial, and cancer therapy.^[^ ^74^ ^]^ In addition to the inherent bioactivity of metal ions and organic linkers, the advantages of large surface area and functional groups also enable MOFs to load drugs, growth factors, and therapeutic gas.^[^ ^75^ ^]^ It should be emphasized that current synthetic approaches of MOFs are mainly carried out through hydrothermal or solvothermal methods; thus, it is necessary to ensure the complete removal of organic reagents to ensure the biosafety of MOFs in vivo. It is also encouraged to develop novel green synthesis approaches to prepare MOFs, which would be more acceptable in biomedical applications.

### 2.5 Piezoelectric Materials

Piezoelectric materials are a type of smart‐responsive materials that can achieve the conversion of electrical energy and mechanical energy through electromechanical coupling effects.^[^ ^76^ ^]^ Piezoelectric materials have two types, including positive piezoelectric effects that generate charge under mechanical stress, and inverse piezoelectric effects that make materials deform under electrical fields. From the perspective of material systems, piezoelectric materials can also be divided into organic, inorganic, and composite piezoelectric materials; their properties and biomedical application can be found in previous review articles. In this part, we are mainly focusing on inorganic piezoelectric materials owing to their advantages of high piezoelectricity, high electromechanical coupling coefficients, and good biocompatibility.

At present, lead‐based piezoelectric ceramics are extensively applied in medical devices such as medical imaging and ultrasound diagnosis owing to its high piezoelectricity that can reach 2000 pC N^−1^.^[^ ^77^ ^]^ However, the potential cytotoxicity of lead elements attracts researchers’ concerns and limits their application as an implant. Therefore, lead‐free inorganic piezoelectric materials including barium titanate (BaTiO_3_, BTO), strontium titanate (SrTiO_3_), potassium sodium niobate (K_0.5_Na_0.5_NbO_3_, KNN), and zinc oxide (ZnO) are developed, which exhibit good piezoelectricity and biocompatibility. These piezoelectric materials can be implanted in vivo to exert their long‐term biological effects such as bone regeneration, wound healing, and nerve regeneration.^[^ ^76^, ^78^ ^]^ The mechanical stress of piezoelectric materials can originate from body movement or external stimulations such as ultrasound and magnetic fields. BTO is a typical perovskite structured piezoelectric material, which has high piezoelectric coefficient (d_33_) reaching at 190 pC N^−1^.^[^ ^12a^ ^]^ It is reported that BTO has well biocompatibility and would not affect cell viability even at high concentrations.^[^ ^79^ ^]^ More importantly, the piezoelectric properties of BTO can be easily modulated through doping single/multi metal elements such as Zr, Sr, and Ca, and modified with other functional materials, which obviously broaden its application scenarios.^[^ ^80^ ^]^ For example, Au nanoparticles modified‐BTO nanoparticles were developed to generate H_2_ via piezoelectric catalysis under ultrasound, which enabled scavenging the excess ROS of the injured spinal cord to alleviate its inflammatory response.^[^ ^81^ ^]^ ZnO is another widely used piezoelectric material in biomedicine with wurtzite structures.^[^ ^82^ ^]^ Although their piezoelectric coefficient (d_33_) is obviously lower than that of BTO, the electrical signals generated by ZnO are enough to influence cell membrane potential; thus, regulating cell fates.^[^ ^83^ ^]^ In a recent study, ZnO‐based nerve guidance conduits were developed for the treatment of peripheral nerves regeneration. Piezoelectric signals generated by the scaffolds effectively promoted the myelination‐related genes expression of Schwann cells and induced axon growth, thereby restoring its motor functions.^[^ ^84^ ^]^

## 3 Inorganic‐Based Material Composites for Innervated Tissue Regeneration

To date, a series of inorganic biomaterials itself or combined with polymer matrixes to form inorganic‐based material composites and have been applied for nerves and nerve‐innervated tissue regeneration with functional recovery. In the following sections, we mainly introduce the rational design and characteristics of four types of inorganic‐based material composites including inorganic biomaterial‐containing membranes, inorganic biomaterial‐containing hydrogel, 3D printing of inorganic biomaterial‐based scaffolds, and 3D bioprinting of inorganic biomaterial‐based cellular constructs; their suitable applications will also be discussed (Figure 2).

### 3.1 Inorganic Biomaterial‐Containing Membranes

Due to the advantages of flexibility, softness, processability, and tunable micro/nano structures, membranes have been widely used in soft tissue regeneration, including skin, peripheral nerves, cardiac tissues, and so on.^[^ ^85^ ^]^ Membranes have porous structures which could provide biomimetic microenvironment for cell adhesion. Up to now, various methods including electrospinning, layer‐by‐layer deposition, and vacuum filtration‐assisted self‐assembly have been used to prepare inorganic biomaterials‐containing membranes.^[^ ^86^ ^]^ The incorporation of inorganic biomaterials into polymers can not only modulate its physiochemical properties, such as hydrophilicity and mechanical strength, but also serve as bioactive agent to improve its bioactivity.

It was known that the hydrophilicity of scaffolds would affect cell attachment and protein absorption. The contact angle analysis showed that synthetic polymers polylactic acid (PLA) membrane was hydrophobic with the contact angle of 91.96° ± 7.06°; while, the incorporation of hardystonite bioceramic (ZnCS, Ca_2_ZnSi_2_O_7_) particles greatly improved its hydrophilicity with the contact angle of 2.34° ± 0.32°, contributing to the wound exudate absorb capacity and cell adhesion.^[^ ^87^ ^]^ To enhance its bioactivity, the strategies of incorporating inorganic bioactive materials into various polymers to fabricate composite membranes have been extensively studied. Bioceramics embedded in the fibers could inhibit the sudden release of ions, which greatly avoided the potential inflammatory reactions. Spindle zinc silicate (Zn_2_SiO_4_) nanoparticles were added into polycaprolactone (PCL) to prepare wound dressing for the treatment of deep burns via electrospinning.^[^ ^88^ ^]^ The released Zn and Si ions endowed the membranes with improved neurogenic and angiogenic bioactivity. In another study, Pi et al. designed a flexible sono‐piezo membrane that was composed of PCL and piezoelectric ZnO nanoparticles via electrospinning for functional skin regeneration.^[^ ^89^ ^]^ Under the low‐intensity pulsed ultrasound (LIPUS, 1 MHz, 0.5 W cm^−2^), the membrane could generate electrical signals to provide pro‐regenerative microenvironments to promote innervation and sweat glands regeneration. In addition to wound healing, inorganic biomaterials‐based membranes also acted as myocardial patches for the repair of myocardial infarction.^[^ ^90^ ^]^ Magnetostrictive CoFe_2_O_4_ nanoparticles were incorporated into piezoelectric polyvinylidene fluoride (PVDF) nanofibers to form myocardial patch by using electrospinning. Under magnetic field, the patch could provide magnetoelectric cues to synergistically repair the damaged cardiomyocytes.

Moreover, owing to its easy processability, inorganic biomaterial‐containing membranes could also be programmed to be processed into various 3D macrostructures by rolling, cutting, and folding, obviously broadening its application scenarios. For example, piezoelectric KNN was incorporated into PLA to prepare aligned nanofiber membranes by using electrospinning, which subsequently rolled up to obtain 2 mm cylindrical multichannel 3D scaffolds for spinal cord regeneration.^[^ ^78b^ ^]^ Under ultrasound irradiation, 3D scaffolds could generate electrical signals to promote the neuronal differentiation of NSCs and angiogenesis. Similarly, Sun et al. introduced Li–Mg–Si bioceramic into PCL fibers to prepare composite membranes via electrospinning, and then, rolled them up to form nerve guidance conduits (NGCs) with length of 12 mm and inner diameter of 2 mm for peripheral nerve regeneration.^[^ ^91^ ^]^

### 3.2 Inorganic Biomaterial‐Containing Hydrogel

Hydrogel has high water content and porous networks that are suitable to mimic the natural extracellular matrix microenvironments.^[^ ^92^ ^]^ Hence, hydrogel‐based biomaterials have been widely used in tissue engineering and regenerative medicine. From the source of raw materials, hydrogel can be divided into synthetic and natural hydrogels. Synthetic hydrogels including polyvinyl alcohol (PVA), PLA, and poly(ethylene glycol) (PEG) have tunable mechanical strength, rheology, and degradation properties, making them widely applicable to both hard and soft tissues regeneration. However, the potential residues of toxic additives and monomers need to be carefully considered.^[^ ^93^ ^]^ Besides, synthetic hydrogels lack cell adhesion site, which leads to the relatively poor biocompatibility and bioactivity. The incorporation of inorganic biomaterials into synthetic hydrogel is a promising approach to improve its biocompatibility and enhance cell–materials interactions.^[^ ^94^ ^]^ On the other hand, natural hydrogels are usually derived from polysaccharides (e.g., alginate, chitosan, and hyaluronic acid), proteins(e.g., collagen and silk), and their derivatives(e.g., gelatin).^[^ ^95^ ^]^ Due to the inherent structural similarity with native tissues, natural hydrogels possess excellent biocompatibility, bioactivity, and biodegradability, making them to be extensively applied for tissue regeneration. However, the inferior mechanical properties and potential immunological rejection of natural hydrogels limit their applications to some extent.

At present, various inorganic biomaterials are integrated into polymer matrixes to prepare the organic–inorganic composite hydrogel for biomedical application.^[^ ^96^ ^]^ The introduction of inorganic biomaterials could improve its mechanical properties and bioactivity. It is known that bioceramic has the capacity to release multiple ions to regulate cell behaviors; while, the early fast ionic release profile may induce inflammatory response, attracting more attentions. The incorporation of bioceramic into hydrogel avoids the direct contact with body fluids, which largely alleviates the rapid release of ions. As an example, Sr‐doped calcium silicate nanowires (Sr‐CSH) were introduced into gelatin methacryloyl (GelMA) matrix to form nanocomposite hydrogel. The composite hydrogel achieved a sustained release of Sr and Si ions for 14 days, which contributed to long periods participation of bone regeneration.^[^ ^97^ ^]^ Besides, the incorporation of Sr‐CSH also enhanced its mechanical strength for more than two times, as compared with pure GelMA hydrogel that was suitable for osteogenic differentiation of BMSCs. Moreover, the interaction of inorganic biomaterials and polymer matrix also affects its physicochemical properties. For example, nanoengineered ionic covalent entanglement hydrogel is composed of electrostatically charged 2D nanosilicates, kappa‐carrageenan (kCA), and GelMA.^[^ ^98^ ^]^ The surface charges of nanosilicates can bind with polymers to form noncovalent electrostatic bonds, which contribute to increased compressive strength and toughness. Besides, 2D nanosilicates also improve the mineralization and osteoinductive capacity. In another research, amine‐functionalized mesoporous bioactive glass nanoparticles (AMBGN) were combined with gelatin and oxidized dextran to form flexible osteogenic glue for fracture bone repair.^[^ ^99^ ^]^ The dynamic covalent bond between amine group of AMBGN and aldehyde group of oxidized dextran endowed the composite hydrogel with favorable injectability and self‐healing properties. More importantly, the addition of AMBGN significantly enhanced the compressive strength and adhesive strength, which enabled to activate YAP signals pathway for stimulating osteogenesis. Moreover, inorganic biomaterials can also act as crosslinking agents to crosslinking polymers to form composite hydrogel. As an example, an injectable macroporous hydrogel composed of bioactive glass (BG), methacrylated hyaluronic acid, and 3‐aminophenylboronic acid‐modified sodium alginate was applied for cell delivery.^[^ ^100^ ^]^ The microgels were first obtained by squeezing the bulk photocrosslinked hydrogel through steel meshes, and then, these microgels were mixed with BG particles. The rapid release of ions from BG could increase pH to create an alkaline microenvironment, which further triggered the formation of a dynamic B─O bond between boric acid and hydroxyl groups; thus, leading to the self‐assembly of these microgels to form macroporous hydrogel. The presence of BG promoted angiogenesis, and its macroporous network structures induced cell infiltration, which synergistically accelerated tissue regeneration.

In addition, inorganic biomaterials can also endow composite hydrogel with smart responsive properties to expand its application field and facilitate clinical convenience. Upon external stimulation such as light, ultrasound, and magnetic stimuli, the composite hydrogel can generate heat, therapeutic gas, and electrical currents; thus, achieving multimodal therapy, shape change, and controllable drug release.^[^ ^101^ ^]^ For example, β‐FeSi_2_ particles were added into alginate matrix to fabricate sprayable hydrogel for both tumor therapy and wound healing.^[^ ^102^ ^]^ β‐FeSi_2_ particles served as therapeutic agents that endowed the hydrogel with excellent photothermal and chemodynamic effects for suppressing tumors; what's more, the released Fe and Si ions promoted angiogenesis and wound healing. In another study, light responsive multifunctional hydrogel containing DFO‐loaded BP nanosheet, GelMA, and Alg‐MA was prepared for bone regeneration.^[^ ^103^ ^]^ Under NIR irradiation, the hydrogel could generate a mild photothermal microenvironment and trigger the release of DFO and PO_4_ ions, which synergistically promoted vascularized bone regeneration. Moreover, the generated heat triggered by external stimuli would also affect the structure of hydrogel, leading to controllable swelling or shrinkage behavior. Thermosensitive hydrogel composed of MXene‐modified magnetic nanoparticles, poly(N‐isopropyl acrylamide) (PNIPAM), and alginate were utilized for infected wound healing.^[^ ^104^ ^]^ PNIPAM is a temperature sensitive polymer and shrinks when the temperature is higher than the lower critical solution temperature. The increased temperature induced by external NIR or alternating magnetic field (AMF) stimuli could trigger the shrinkage of composite hydrogel, leading to the release of Ag nanoparticles for bacteria elimination. The external stimuli induced controllable release of therapeutic gas including O_2_, H_2_, and NO plays an important role in regulating cell behaviors.^[^ ^42^, ^105^ ^]^ Ultrasound responsive piezoelectric Au‐functionalized BaTiO_3_ nanoparticles (Au@BTO) were integrated with chitosan hydrogel for spinal cord repair.^[^ ^81^ ^]^ Under ultrasound stimuli, the generated H_2_ from composite hydrogel could scavenge ROS of the injury site to restore the redox balance and protect neurons from apoptosis, thereby contributing to the recovery of spinal cord injury.

### 3.3 3D Printing of Inorganic Biomaterial‐Based Scaffolds

During the past few decades, 3D printing is an advanced additive manufacturing technique widely used in tissue engineering and regenerative medicine. 3D printing has the advantages of precisely controlling the spatial distribution of biomaterials, growth factors, and even of living cells via layer‐by‐layer deposition, which could highly mimic the hierarchical structure and composition of native tissues, which is much more challenging for traditional methods. The major commonly used 3D printing techniques include extrusion, stereolithography (SLA), and selective laser sintering (SLS) printing. Besides, the combination of different 3D printing approaches also helps to fabricate scaffolds with complex structures. The summary of advantages and limitations of these techniques can be found in previous review articles.^[^ ^106^ ^]^

Owing to its excellent biocompatibility and bioactivity, inorganic biomaterials have been directly used or combined with other materials for 3D printing to fabricate tissue regenerative scaffolds. Inorganic biomaterials themselves, including bioceramic, metals, and their alloys have been directly used to prepare scaffolds through extrusion, DLP, and SLS‐based 3D printing techniques.^[^ ^107^ ^]^ The spatial structure, compressive strength, pore size, and porosity can be precisely controlled via programmed design. Moreover, inspired by the special structures (e.g., brick‐and‐mortar, plant blade, lotus, and gear) in nature, our groups have developed several biomimetic bioceramic scaffolds for tissue regeneration, including lotus root‐like bioceramic scaffolds for vascularized bone regeneration,^[^ ^108^ ^]^ tree‐like bioceramic scaffolds for innervated bone regeneration,^[^ ^32^ ^]^ and polyhedron‐like bioceramic scaffolds for neuro‐vascularized bone regeneration.^[^ ^109^ ^]^ In addition, 3D printing could also combine with other approaches to fabricate hierarchical structural scaffolds. As an example, Li et al. combined extrusion 3D printing and bidirectional freezing to fabricate hot‐dog‐like bioceramic scaffolds composed of hollow bioceramic tubes embedded with aligned lamellar bioceramic rods.^[^ ^33b^ ^]^ This hierarchical structural scaffold possessed favorable drug loading and releasing behaviors; thus, promoting BMSCs differentiation. In another study, Yu et al. developed gear‐like bioceramic scaffolds with aligned microgroove structures via needle‐modified extrusion 3D printing.^[^ ^110^ ^]^ The well‐ordered surface microstructures exhibited good immunomodulatory properties for regulating macrophages M2 polarization and enhancing the osteogenic differentiation of BMSCs. In addition to bioceramic, metal and their alloys were also used to prepare 3D‐printed orthopedic scaffolds and cardiovascular stents.^[^ ^111^ ^]^ For example, Mg–Nd–Zn–Zr alloy implants with suitable mechanical strength were prepared by using SLS‐based printing for the treatment of infectious bone regeneration.^[^ ^112^ ^]^ The released bioactive ions endowed the scaffolds with good anti‐bacterial, anti‐inflammatory, and osteo‐inductivity properties.

In addition to directly printing inorganic materials, inorganic biomaterials could also serve as bioactive agents and be integrated with other materials to improve the bioactivity of 3D printed composite scaffolds. Functional inorganic biomaterials endow 3D printed scaffolds with novel properties such as osteogenesis, angiogenesis, neurogenesis, anti‐bacterial, anti‐oxidant, and anti‐tumor. For example, our group proposed the idea of developing bifunctional scaffolds for both tumor therapy and tissue regeneration. A common strategy was incorporating therapeutic inorganic biomaterials into 3D printed scaffolds.^[^ ^113^ ^]^ For example, 2D wesselsite nanosheet (SrCuSi_4_O_10_) with well NIR‐II photothermal conversion properties was mixed with PCL to print 3D composite scaffolds.^[^ ^114^ ^]^ The generated hyperthermal of composite scaffolds via NIR‐II light effectively killed Saos‐2 cells and ablated osteosarcoma in vivo. Besides, the released bioactive Sr, Cu, and Si ions also improved the angiogenic and osteogenic activity of composite scaffolds and contributed to enhanced vascularized bone regeneration. In another study, 3D printed bifunctional bioactive scaffolds were prepared through integrating Fe_3_O_4_ microspheres and calcium silicate nanowires into GelMA matrix.^[^ ^115^ ^]^ Fe_3_O_4_ microspheres under alternating magnetic field showed magnetothermal properties for killing breast tumor cells; while, calcium silicate nanowires enabled to release bioactive ions for inducing the adipogenic differentiation of ADSCs. Moreover, surface functionalization is another effective approach to fabricate bifunctional scaffolds through modifying functional inorganic materials on the surface of 3D printed scaffolds. For example, Gao et al. soaked 3D printed bioceramic scaffolds in Mn single‐atom nanozyme dispersion to prepare composite scaffolds with enhanced anti‐bacterial and osteogenesis properties.^[^ ^116^ ^]^ The scaffolds could generate ROS under ultrasound to eliminate *Staphylococcus aureus* (*S. aureus*) or *Escherichia coli* (*E. coli*), which further promoted osteogenic differentiation of BMSCs. Similarly, other functional inorganic biomaterials including rGO, BP, MXene, and LDH were also integrated with 3D printed scaffolds for designing multifunctional scaffolds.^[^ ^73^ ^]^

### 3.4 3D Bioprinting of Inorganic Biomaterial‐Based Cellular Constructs

3D bioprinting has gained rapid development and is emerging as an advanced approach for the fabrication of biomimetic constructs that enable reconstruction of damaged tissue/organs.^[^ ^117^ ^]^ By using different printing strategies, such as extrusion‐based, inkjet‐based, and DLP‐based bioprinting techniques, bioinks loaded with living cells were precisely deposited in a controllable manner to form 3D cellular constructs. 3D bioprinting integrates the advantages of both cell transplantation therapy and 3D printing technique, specifically manifested in the following points: 1) 3D bioprinting enables to precisely deposit multiple biomaterials with heterogeneous physicochemical properties to mimic the complex gradient structures of native tissues, such as osteochondral, skin, and brain tissues.^[^ ^118^ ^]^ 2) As compared with direct cell injection therapy, 3D bioprinted cellular constructs can not only protect exogenous cells from apoptosis to improve cell viability but also provide the cell adhesion niche to enhance cell retention efficiency.^[^ ^119^ ^]^ 3) The combination of 3D bioprinting and organoids can achieve the mass fabrication of biomimetic tissues/organs, which is expected to solve the problem of donor site shortage of autologous transplantation. More excitingly, with the continuous approval of cell transplantation therapy‐related products in the clinical setting, it is believed that more 3D bioprinted cellular constructs will enter the clinical trial stages in the coming years.

With regards to fabrication of functional cellular constructs, the design of bioinks is of pivotal importance.^[^ ^120^ ^]^ Ideal bioinks should possess several crucial properties as the following.^[^ ^121^ ^]^ First, suitable biocompatibility is the fundamental requirement of bioinks, which ensures supporting of the survival of encapsulated cells. Besides, it is worth noting that bioinks should not generate any toxic substances from degradation during the culture periods. Second, good printability is quite essential for maintaining the structure integrity of constructs, especially for the preparation of large‐scale scaffolds. Finally, bioinks should have excellent bioactivities that provide suitable microenvironments for regulating the proliferation and differentiation behaviors of loaded cells. Hence, it is now quite challenging to design bioinks that meet all the above requirements. Commonly, researchers use different types of hydrogels as bioinks for the fabrication of cell‐laden scaffolds.^[^ ^122^ ^]^ As described previously, although natural hydrogel including collagen, decellularized extracellular matrix, and silk fibroin has good biocompatibility and biodegradability, it suffers from poor mechanical properties and structural stability. On the contrary, although synthetic hydrogels such as PVA, PCL, and PEG possess tunable mechanical properties, it is faced with the question of relatively poor biocompatibility compared with natural hydrogel due to the lack of cell‐binding sites and potential toxic additives or residuals. In addition, it should be emphasized that hydrogel itself usually does not have the ability to regulate specific differentiation of encapsulated cells. Thus, various growth factors such as BMP‐2, VEGF, and NGF were added into bioinks to modulate cell behaviors, but they faced the problems of expensive prices and easy inactivation during the bioprinting process.

In recent years, researchers attempt incorporating inorganic biomaterials into bioinks to improve its physiochemical and biological properties. When inorganic biomaterials are mixed with hydrogels, the morphology, size, surface charge, and other characteristics of inorganic biomaterials will affect the rheological and mechanical properties of bioinks. For example, Zhang et al. previously incorporated calcium silicate nanowires into GelMA hydrogel to form inorganic‐based bioinks for 3D bioprinting of neuro‐bone multicellular constructs.^[^ ^123^ ^]^ The addition of calcium silicate nanowires could decrease its yield stress (intersection of storage modulus and loss modulus), making the bioinks be more smoothly extruded from the needles, improving its printability. Besides, the incorporated calcium silicate nanowires also improved the mechanical strength of the constructs. Moreover, the functional group, surface charge, and released bioactive ions of inorganic biomaterials could interact with the functional groups of hydrogels through electrostatic interaction and ionic bonding, further contributing to improved printability and mechanical properties. As an example, Lee et al. incorporated amine‐modified silica nanoparticles (Am‐SiNPs) into oxidized alginate (OSA) and gellan gum hydrogel networks to form nanocomposite bioinks.^[^ ^124^ ^]^ The amine groups of Am‐SiNPs could interact with the aldehydes group of OSA to form reversible imine bonds, which improved the rheological behaviors, mechanical strength, and structural fidelity. In another study, 2D nanosilicate clay (Laponite) was incorporated with glycosaminoglycan nanoparticles (GAGNPs) to form composite bioinks.^[^ ^125^ ^]^ The electrostatic interaction between the negative charge of GAGNPS and positive charge of Laponite endowed the bioinks with tunable rheological and mechanical properties. The nanoengineered composite bioinks enabled to fabricate complex structures with high shape fidelity without an additional crosslinking process.

More importantly, inorganic biomaterials can serve as safe and effective bioactive agents to enhance the biological activity of encapsulated cells within bioprinted scaffolds. Inorganic biomaterials can provide biochemical and biophysical cues for modulating cell–materials and cellular interactions. It is known that inorganic biomaterials have the capacity to release multiple ions (e.g., Ca, Mg, Si, Mn, Li, and Sr) to create suitable ionic microenvironment, which is beneficial to inducing cell proliferation and differentiation. During the past few years, our groups have combined silicate biomaterials with 3D bioprinting to developing a series of silicate bioinks‐based cellular constructs for complex tissues regeneration.^[^ ^121^ ^]^ As an example, Li–Mg–Si bioceramic was combined with hydrogel as inorganic bioinks for 3D bioprinting of biomimetic multicellular scaffolds for osteochondral regeneration.^[^ ^27^ ^]^ The ionic microenvironment created by the released Li, Mg, and Si ions could simultaneously promote the osteogenic differentiation of loaded BMSCs and maturation of chondrocytes. Similarly, strontium silicate microparticles‐containing bioinks were used to fabricate human umbilical vascular endothelial cells (HUVECs)–human dermal fibroblasts (HDFs) multicellular scaffolds via the combination of extrusion and inkjet bioprinting.^[^ ^126^ ^]^ The sustained release of Sr and Si ions enhanced the angiogenic activities of HUVECs and promoted vascularized skin regeneration in vivo. Recently, immunomodulatory‐based strategies have been regarded as a promising approach for tissue regeneration. Remodeling the immune microenvironment of the injury area can enhance the bioactivities of cells. Recently, Du et al. developed an immunomodulatory multicellular scaffold based on manganese silicate (MS)‐containing bioinks for tendon‐to‐bone regeneration.^[^ ^127^ ^]^ The released Mn ions exhibited remarkable immunomodulatory properties for inducing macrophages polarization toward M2 phenotypes, thereby promoting the tenogenic differentiation of tendon stem/progenitor cells (TSPCs) and osteogenic differentiation of BMSCs. Moreover, the introduction of electroactive biomaterials such as GO, BP, and MXenes into bioinks could help to rebuild the electric microenvironments; even when combined with external stimuli, they could better promote tissue regeneration. These strategies have been used in spinal cord regeneration, bone regeneration, and wound healing.

In addition, inorganic biomaterials within bioinks could also provide topographical cues for regulating cell behaviors. As an example, Au nanowires‐containing bioinks were mixed with C2C12 cells to fabricate muscle constructs.^[^ ^128^ ^]^ Appropriate shear pressure during the bioprinting process and applied electrostatic field after bioprinting could synergistically induce the alignment of Au nanowires within the bioinks, which effectively promoted the myogenic differentiation of C2C12 cells and myotube formation. To enhance the survival and metabolism activity of loaded cells with the scaffolds, typical O_2_ generating inorganic biomaterials calcium peroxide (CPO) were incorporated into GelMA hydrogel for 3D bioprinting.^[^ ^105^ ^]^ The sustained generation of O_2_ from CPO particles obviously improved the viability and metabolic activity of encapsulated cells under hypoxic conditions, as compared with the CPO‐free scaffolds.

## 4 Application in Innervated Tissue Regeneration and Functional Recovery

Due to their exceptional neurogenic, osteogenic, angiogenic, and immunomodulatory bioactivities, inorganic biomaterials are extensively used for tissue engineering and regenerative medicine. In this section, we will introduce the broad applications of inorganic biomaterials in innervated multi‐tissue regeneration, such as central nerves, peripheral nerves, bone, skin, and skeletal muscles. The detailed summary of inorganic biomaterials and their beneficial effects on nerves and nerve‐innervated tissue regeneration are listed in **Table** **1**.

**Table 1:** Summary of inorganic‐based material composites and their effects on nerves and nerve‐innervated multi‐tissue regeneration.

Tissue/organ	Inorganic components	Material composites	Biological effects	Refs.
Central nerves	HAp nanorods	Injectable hydrogel	Neuronal differentiation of NSCs	[138]
	GeP nanosheet	Hydrogel	Neuronal differentiation of NSCs, immune regulation, and angiogenesis	[133]
	MnO_2_ nanoparticles	MSCs‐laden hydrogel	Neural differentiation of MSCs and anti‐oxidant	[43a]
	Laponite	Injectable hydrogel	Promoting axonal growth and inhibiting glial scars	[135]
	K_0.5_Na_0.5_NbO_3_, KNN	Fibrous scaffolds	Neuronal differentiation of NSCs and angiogenesis	[78b]
	MWCNTs	Membranes	Neural differentiation of MSCs	[141]
	MgO	Fibrous scaffolds	Anti‐apoptosis and neuronal differentiation of NSCs	[136]
	Ti_3_C_2_T*_X_* MXenes	Injectable hydrogel	Angiogenesis, remyelination, and axon regeneration	[128]
	Ti_3_C_2_T*_X_* MXenes	Injectable hydrogel	Neuronal differentiation of NSCs, axonal growth, myelin regeneration, and reducing glial scar deposition	[134]
	BP nanosheet	—	Neural differentiation of neural progenitor cells	[139]
	Au@BTO nanoparticles	Injectable hydrogel	Anti‐inflammatory and anti‐apoptosis	[81]
	MgAl‐LDH	—	Neuronal differentiation of NSCs and immune regulation	[131]
	MgMn‐LDH	Injectable hydrogel	Scavenging ROS, neurite growth, and differentiation	[72]
	Graphene nanosheet	—	Neuronal differentiation of NSCs	[144]
Peripheral nerves	Ti_3_C_2_T*_X_* MXenes	4D printed scaffolds	SCs proliferation and migration, myelin regeneration, and angiogenesis	[150]
	Ca_7_MgSi_4_O_16_ bioceramic	Fibrous scaffolds	SCs myelination, immune regulation, and angiogenesis	[149]
	Li_2_MgSiO_4_ bioceramic	Fibrous scaffolds	SCs myelination, immune regulation, and angiogenesis	[91]
	CNTs	Hydrogel	Axonal outgrowth of neurons	[78a]
	Mg ions	Hydrogel	Neurite outgrowth, axon regeneration, and myelination	[147]
	SiP@PDA nanosheet	Hydrogel	Myelination, axonal regeneration, immunoregulation, and vascularization	[148]
	rGO	3D bioprinted hydrogel scaffolds	SCs proliferation and differentiation	[155]
	rGO, ZnO	Microneedles scaffolds	SCs migration and myelination, inhibiting muscle atrophy	[84]
	BTO	Fibrous hydrogel	Neuronal‐oriented extension and neurite outgrowth	[152]
	Fe(acac)_3_ nanoparticles	Hydrogel	Production of SCs‐derived EVs, contribute to axon growth, angiogenesis, and inflammatory regulation	[46]
	NaYF_4_:Yb,Tm@NaYF_4_ UCNPs	—	Promoting proliferation, secretion of nerve growth factor, and neural function of SCs	[154]
Bone	Silicified‐collagen	Fibrous scaffolds	Inducing axon outgrowth and expression of Sema3A and Sema4D of DRG	[24]
	Ca_18_Mg_2_(HPO_4_)_2_(PO_4_)_12_	Hydrogel	Neurogenesis and osteogenesis	[226]
	BP@Mg nanosheets	Hydrogel	Neurogenesis, angiogenesis, and osteogenesis	[67]
	rGO	3D printed hydrogel scaffolds	SCs myelination and osteogenesis	[164]
	Mg ions, Zn‐doped bioglass	3D printed scaffolds	Neurogenesis, angiogenesis, and osteogenesis	[227]
	Zn‐Ca_18_Mg_2_(HPO_4_)_2_(PO_4_)_12_	Membranes	Neurogenesis, angiogenesis, and osteogenesis	[165]
	Calcium silicate nanowires	3D bioprinted multicellular constructs	Neurogenic differentiation of SCs and osteogenesis	[123]
	Mo_2_Ti_2_C_3_ nanosheet	Hydrogel	Neurogenesis and osteogenesis	[163]
	Cu–GeP nanosheet	Hydrogel	Neurogenesis, angiogenesis, osteogenesis, and anti‐bacterial	[68]
	Li–Ca–Si bioceramic	3D bioprinted cellular constructs	Neurogenesis, angiogenesis, and osteogenesis	[140]
	TCP	3D printed tree‐like bioceramic scaffolds	Neurogenesis and osteogenesis	[32]
	TCP	3D printed polyhedron‐like bioceramic scaffolds	Neurogenesis, angiogenesis, and osteogenesis	[109]
Dental tissue	Ca–Mg–Si bioceramic	Injectable microspheres	Enhancing secretion of CGRP of neurons and osteogenesis	[179]
Skeletal muscles	Au nanowires	3D bioprinted myoblast‐laden constructs	Cell alignment and myogenesis	[128]
	CaSiO_3_, SrCO_3_	Injectable hydrogel	Myogenesis, inhibiting muscle necrosis, inducing macrophage M2 polarization, and angiogenesis	[190]
	SiO_3_^2−^ ions	hydrogel	Myoblast proliferation and myogenic differentiation, angiogenesis	[189]
	CNT, Fe_3_O_4_	Injectable nanofibers hydrogel	Inducing aligned myofiber formation	[188]
	GO	3D bioprinted myoblast‐laden constructs	Promoting myogenic differentiation of myoblasts	[187]
Tendon	MoS_2_ nanosheet	Neuromorphic patch	Promoting noradrenaline release and anti‐inflammation	[195]
Skin	Graphene	Self‐power membrane patch	Neurogenic differentiation of MSCs	[200]
	Cu–calcium silicate	Fibrous membrane	Neural networks reconstruction, angiogenesis, and hair follicle regeneration	[205]
	ZnO	Fibrous membrane	Innervation, angiogenesis, and functional sweat gland repair	[89]
	BG@Cu + Mg	Hydrogel	Neurogenesis, angiogenesis, and anti‐microbial	[204]
	Mg–gallic acid MOFs	Sprayable hydrogel	Anti‐inflammatory, anti‐bacterial, neurovascular networks reconstruction, and re‐epithelialization	[206]
	Zr‐MOFs (NU‐801)	Hydrogel	Sensory recovery and hair follicle neogenesis	[208]
	Zn–curcumin MOFs	Hydrogel	Anti‐inflammatory, anti‐bacterial, neurogenesis, and angiogenesis	[207]
	TiO_2_/Bi_2_S_3_ nanotubes	—	Fibroblast activation, neural differentiation, and immune modulation	[201]
	Zn_2_SiO_4_ nanoparticles	Membranes	Neurogenesis and angiogenesis	[88]
	MgSiO_3_ nanoparticles	Sprayable hydrogel	Neurogenesis, angiogenesis, and immune modulation	[203]
Corneal tissue	CeO_2_	—	Anti‐oxidation, anti‐inflammation, anti‐apoptosis, anti‐angiogenesis, and neuroprotection	[213]
Cardiac tissue	PEDOT nanoparticles	Hydrogel	Anti‐inflammation, angiogenesis, and cardiac nerve remodeling	[219]
Cavernous tissue	CNT	Sprayable hydrogel	Promoting the neuronal differentiation of neural stem cells, inducing axon outgrowth of DRG neurons, and immune modulation	[223]
	BTO, GO	Membranes	Stimulating the proliferation and differentiation of SCs, promoting axonal growth and myelination	[224]
	BP	—	Recruiting the endogenous stem cells	[225]

### 4.1 Central Nerves

The central nervous system comprises brain and spinal cord, is the commander of the human body that controls the physical and mental functions.^[^ ^3b^ ^]^ Central nerves injury would destroy the neural networks, leading to the loss of sensory and motor functions. Hence, central nerves injury remains the most challenging problem in clinical settings at present. Generally, the harsh microenvironment (e.g., hypoxia, ischemia, oxidative stress, and excessive ROS accumulation) and inherent poor regenerative capacity of neurons are the main obstacles for neural regeneration and functional recovery.^[^ ^129^ ^]^

**Figure 3:**
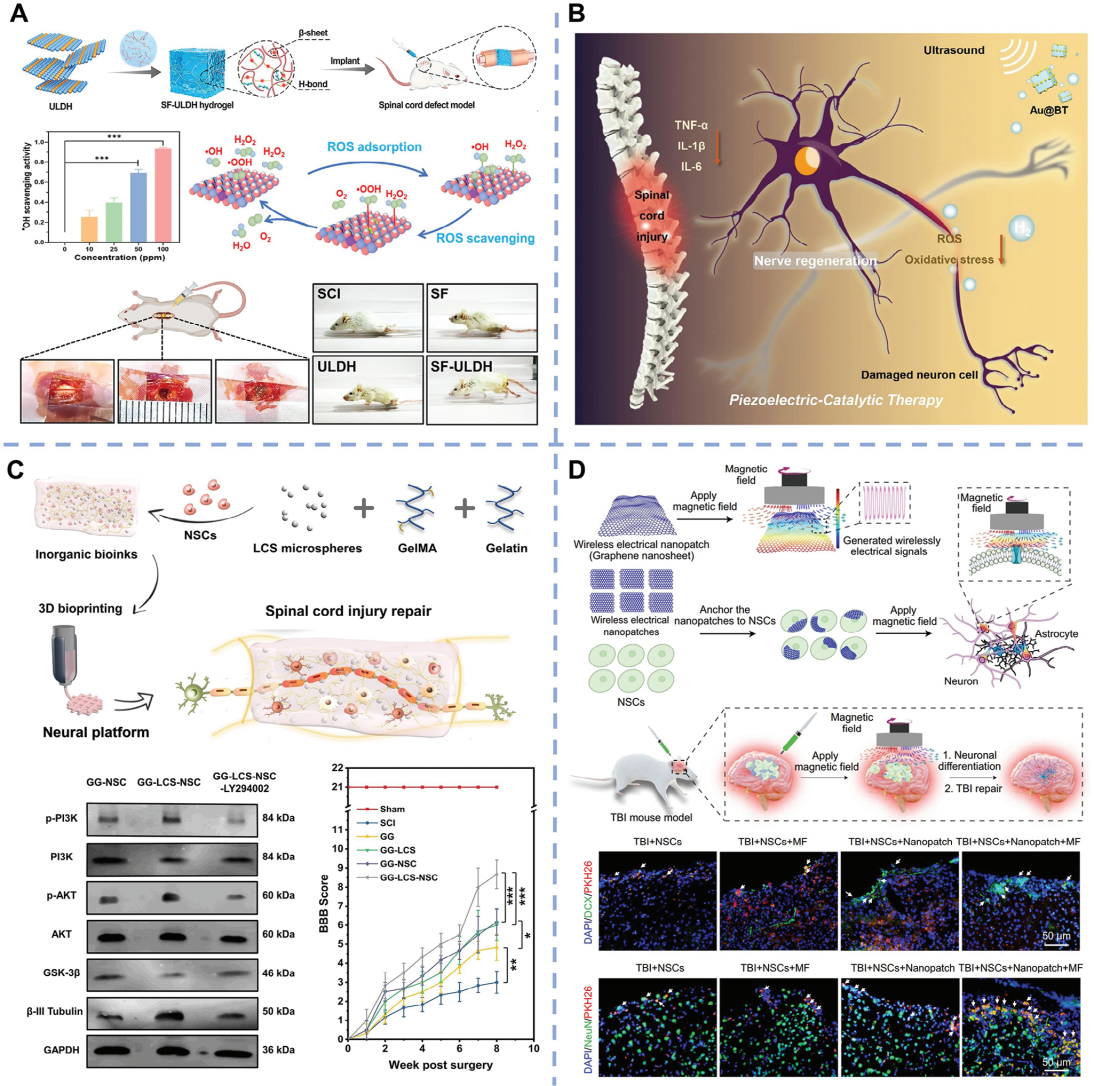
Inorganic biomaterials used for treating central nerves injuries including spinal cord injury and traumatic brain injury therapy. A) Schematic diagram of the design and application of self‐assembled injectable hydrogel containing MgMn‐LDHs and silk fibroin. The released Mn ions enable continuously scavenging ROS and generate O_2_ to alleviate inflammatory environments; while, the released Mg ions promote neurite growth and neuronal differentiation. Reproduced with permission.^[^ ^72^ ^]^ Copyright 2023, Elsevier. B) Au@BT nanoparticles with ultrasound‐triggered piezoelectric‐catalytic generation of H_2_ properties for the treatment of spinal cord injury. Reproduced with permission.^[^ ^81^ ^]^ Copyright 2024, Wiley‐VCH GmbH. C) 3D bioprinting of Li–Ca–Si bioceramic/NSCs constructs for spinal cord repair. Li–Ca–Si bioceramic promoted the neuronal differentiation of NSCs via activating PI3K‐AKT signal pathway; thus, recovering the motor functions of rats. Reproduced with permission.^[^ ^140^ ^]^ Copyright 2024, Oxford University Press. D) Schematic illustration of the wireless graphene nanopatch anchored‐NSCs traumatic brain injury repair. Electrical signals generated by the graphene nanopatch under magnetic field promoted the neuronal differentiation of NSCs. Reproduced with permission.^[^ ^144^ ^]^ Copyright 2024, Springer Nature.

In terms of modulating microenvironments, MnO_2_ and CeO_2_ nanoparticles have been introduced into hydrogels to exert its inherent anti‐inflammatory and antioxidant functions through scavenging the excessive ROS, in turn, improving the bioactivity of neurons. It is reported that spinal cord injury (SCI) would induce the deficiency of Mg ions; while, the supplement of Mg ^would^ decrease neurons apoptosis.^[^ ^130^ ^]^ An injectable self‐assembled hydrogel composed of MgMn‐LDH nanosheet and silk fibroin was used for SCI repair (**Figure** **3**A).^[^ ^72^ ^]^ On the one hand, the released Mg ions had neuroprotective effects and promoted the neurite outgrowth of PC12 cells. On the other hand, the released Mn ions could scavenge ROS and generate O_2_ to alleviate the oxidative stress and hypoxia. In another study, Mg/Al‐LDH had been proved to inhibit M1 marker expression and promote M2 marker expression of microglia; thus, effectively inhibiting inflammatory responses of SCI.^[^ ^131^ ^]^ Moreover, gas therapy is regarded as a safe, green treatment with minimal adverse toxicity to normal tissues/organs, which has been extensively studied in recent years. H_2_, as one of the endogenouses gas, is emerging as an effective anti‐inflammatory agent for the treatment of diabetic wounds, aging bone defects, and neurological diseases.^[^ ^132^ ^]^ H_2_ has many outstanding advantages including the following aspects: 1) H_2_ has high safety and is approved by FDA and China FDA to the list of permitted food additives. H_2_‐rich water is becoming a popular commercial water in China. 2) Due to its high diffusivity, H_2_ can easily cross the blood–spinal cord barrier for the therapy of central nervous system diseases, which is very difficult for traditional drugs or nanomaterials. 3) The byproduct of H_2_ is water, and the excess H_2_ can be excreted by breathing without any residue. In general, these outstanding advantages make H_2_ therapy an emerging therapeutic approach for various biomedical applications. In a recent study, Au nanoparticles‐modified BaTiO_3_ Schottky heterojunction metal–semiconductor composites were prepared and utilized to generate H_2_ via in situ catalyzing H^+^ reduction under ultrasound stimulation (Figure 3B).^[^ ^81^ ^]^ The released H_2_ remarkably scavenged intracellular ROS, as well as promoted the anti‐apoptosis genes expression of Bcl2 and BCL2L1 via the activation of PI3K‐AKT pathway. In vivo results showed that the composites reprogrammed the inflammatory microenvironment of the injury area and recovered the motor functions of SCI rats.

Electroactive materials can simulate the electrophysiological microenvironment of neural tissue, which effectively regulates cell adhesion and differentiation behaviors. Hence, the common strategy is incorporating electroactive materials into hydrogel matrix to obtain conductive hydrogels. Germanium phosphide (GeP), an emerging 2D nanomaterial with suitable conductivity and biodegradability, was added into HA hydrogel for the treatment of SCI.^[^ ^133^ ^]^ The incorporation of GeP nanosheet effectively stimulated the neuronal differentiation of NSCs and promoted the locomotor functions recovery in rat SCI complete transection model. Similarly, an injectable self‐healing hydrogel composed of Ti_3_C_2_T*_x_* MXenes, phytic acid, and polyvinylpyrrolidone was prepared, which provided favorable microenvironment for stimulating angiogenesis, remyelination, and axonal regeneration.^[^ ^134^ ^]^ Further, it is confirmed that the combination of conductive MXenes and electrical stimulation showed better performance of promoting the neuronal differentiation of NSCs; while, inhibiting the formation of astrocytes. This combination strategy ultimately repaired the injured spinal cord tissues in vivo. Not only that, external stimulation could easily adjust the performance of electroactive materials, which is beneficial to build a controllable microenvironment. For example, 3D aligned PLA nanofibers loaded with piezoelectric KNN nanowires could generate controllable timeline, duration, and strength of electrical signals under remote programmed US stimulations. The results showed that piezoelectric nanofibers combined with US stimulation induced the neuronal differentiation of NSCs and angiogenesis; thus, accelerating the locomotor functions recovery.^[^ ^78b^ ^]^

In addition, inorganic biomaterials can serve as delivery platforms in situ deliver drugs and cytokines, such as FGF4, a novel neuroprotective factor. An injectable hydrogel composed of FGF4‐loaded Laponite nanosheet and heparin was designed for preserving and controlling its release.^[^ ^135^ ^]^ The sustained release of FGF4 and bioactive ions showed obvious neuroprotective effect via promoting mitochondrial fusion and modulating mitochondrial localization. In another study, purmorphamine and RA were loaded in mesoporous magnesium oxide (MgO) nanoparticles to enhance the regenerative activity of endogenous cells.^[^ ^136^ ^]^ The released Mg ions could block calcium influx to exert its neuroprotective effects; while, purmorphamine and RA effectively improved the neuronal differentiation activity of NSCs and reduced the formation of glial scars of lesion site.

It is worth noting that the limited number of endogenous NSCs further hinders the effectiveness of the above‐mentioned biomaterials‐based strategies.^[^ ^137^ ^]^ Hence, stem cells transplantation has emerged as a promising approach for SCI repair. However, the low survival and neuronal differentiation efficiency of transplanted NSCs are the major challenges to their therapeutic outcomes.^[^ ^138^ ^]^ Recently, researchers have found that the incorporation of inorganic biomaterials could provide more suitable microenvironment that enhances the activity of transplanted NSCs. BP nanosheet has the capacity of regulating cellular redox homeostasis to promote the neuronal differentiation of NPCs via activating nuclear factor erythroid 2‐like (Nrf2) pathway.^[^ ^139^ ^]^ The transplantation of BP‐loaded NPCs showed higher survival ratio and neuronal differentiation behaviors as compared with pure NPCs. In another study, HAp nanorods enabled to control the fate of NSCs that modulate NSCs, differentiated into mature neurons with electrophysiological behaviors.^[^ ^17a^ ^]^ HAp nanorods were endocytosed into lysosomes; then, the released Ca ions activated the PI3K‐AKT signaling pathway to regulate the neuronal differentiation process. Besides, HAp nanorods were combined with chitosan (CS) and glycerophosphate (GP) to form injectable hydrogels that were used for storage, preservation, and controllable release of NSCs.^[^ ^138^ ^]^ The endocytosis of HAp improved the neuronal differentiation of encapsulated NSCs; thus, contributing to SCI repair in vivo. Moreover, the biophysical aspects of scaffolds also affected cell behaviors because the 3D structure could provide suitable microenvironment for promoting cell–materials and cell–cell interactions. Recently, Zhang et al., developed a 3D bioprinted neural construct through the combination of hydrogels, Li_2_Ca_4_Si_4_O_13_ (LCS) bioceramic and NSCs (Figure 3C).^[^ ^140^ ^]^ The multiple ions released from LCS bioceramic could create suitable ionic microenvironment to promote the proliferation and neuronal differentiation of encapsulated NSCs via activating the PI3K‐AKT signal pathway. The implantation of 3D bioprinted neural constructs effectively promoted axon regeneration and angiogenesis; while, decreasing glial scar formation, thereby promoting SCI repair with functional recovery. In addition, due to the abundant sources of mesenchymal stem cells, inducing MSCs differentiated into neurons is a new approach for neural tissues regeneration, which also helps to alleviate the dilemma of NSCs deficiency. It has been confirmed that MSCs seeded on the surface of multi‐wall CNTs membrane with magnetic field could differentiate into neurons with electrophysiological properties.^[^ ^141^ ^]^

Apart from SCI, traumatic brain injury (TBI) is another leading cause of disability and mortality.^[^ ^142^ ^]^ Due to the poor regenerative ability of neurons, there is currently no effective strategy for the treatment of TBI.^[^ ^143^ ^]^ In a recent study, the authors developed a magnetic field responsive wireless electrical stimulation nano‐patch for TBI therapy (Figure 3D).^[^ ^144^ ^]^ Graphene nanosheets with wireless electrical properties were modified with laminin to tightly anchor NSCs. Under programmed magnetic field, nano‐patch could in situ generate electrical signals to stimulate the wrapped NSCs differentiated into mature neurons via activating MAPK/ERK pathway. The nano‐patch, combined with rotating magnetic field, effectively improved the behaviors and cognitions of TBI mice after implantation for 28 days.

### 4.2 Peripheral Nerves

Peripheral nerves injury caused by trauma and diseases can lead to the loss of movement and sensory dysfunction, which largely affect the mental health and life quality of patients.^[^ ^145^ ^]^ Due to the limited self‐healing ability of nerves, it is urgently required to develop artificial NGCs for the treatment of long‐gap (>8 mm) of nerves injury. Inorganic biomaterials have been incorporated into NGCs to promote peripheral nerves regeneration through modulating immune microenvironment, inducing angiogenesis, enhancing neural cells functions, and increasing bioelectrical conductions.

**Figure 4:**
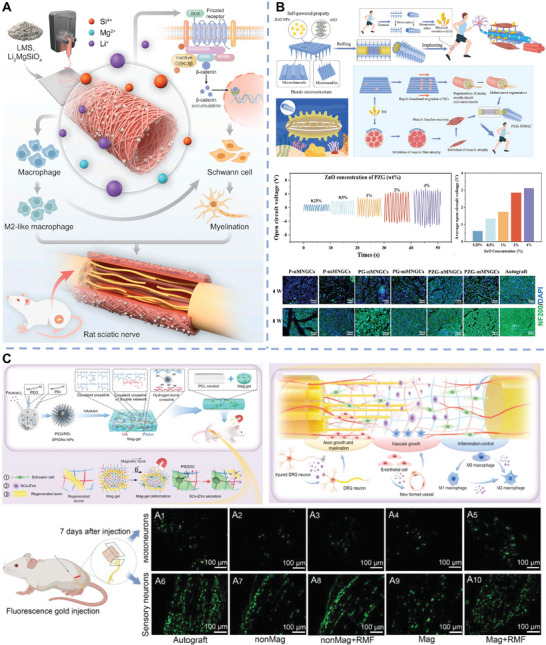
Inorganic biomaterials used for peripheral nerves regeneration. A) Schematic illustration of Li–Mg–Si bioceramic‐based nerve guidance conduits for peripheral nerve regeneration and motor functions recovery. Li–Mg–Si bioceramic can modulate macrophages polarized toward M2 phenotypes and Schwann cell myelination. Reproduced with permission.^[^ ^91^ ^]^ Copyright 2023, Elsevier B.V. B) Schematic representation of microneedle nerve guidance conduit composed of ZnO nanoparticles, rGO, and PCL for preventing muscle atrophy and inducing peripheral nerve regeneration. The conductivity of rGO and piezoelectric properties of ZnO synergistically promoting Schwann cells migration and myelination, as well as restoring neuromuscular functions. Reproduced with permission.^[^ ^84^ ^]^ Copyright 2024, American Chemical Society. C) Superparamagnetic nanoparticles‐functionalized hydrogel scaffolds for enhanced peripheral nerve repair via on‐demand production of Schwann cell's extracellular vesicles. The deformation of magnetic hydrogel under magnetic field can generate micro/nanoscale forces, which trigger the secretion of extracellular vesicles of Schwann cells via activating Piezo signals. Reproduced with permission.^[^ ^46^ ^]^ Copyright 2023, Wiley‐VCH GmbH.

As illustrated above, bioactive ions exhibited favorable bioactivity and represent a promising alternative for peripheral nerves regeneration. Previous studies have found that oral delivery of Mg ions promoted nerves regeneration following crush injury.^[^ ^146^ ^]^ In a study, sustained Mg ions delivery hydrogel was combined with PCL to fabricate NGCs.^[^ ^147^ ^]^ The released Mg ions exhibited remarkable promotion of neurite outgrowth via the activation of PI3K‐AKT pathway and repaired 10 mm nerves defects of rats with motor functions recovery. Moreover, modulating the inflammatory response and providing pro‐regenerative microenvironments are also beneficial to enhance injury‐residue cell functions. A conductive SiP@PDA nanosheet was incorporated into hydrogel to improve its immunomodulatory ability.^[^ ^148^ ^]^ The hybrid hydrogel could provide suitable electrical environment to modulate macrophages polarization, induce the neuronal differentiation of MSCs, and support angiogenesis due to the released Si ions. In another study, bioceramic was incorporated into NGCs to act as bioactive agents for peripheral nerves regeneration (**Figure** **4**A).^[^ ^91^ ^]^ The released Li, Mg, and Si ions effectively modulated M2 macrophages polarization to provide a pro‐regenerative microenvironment and enhanced the proliferation and myelination of SCs via activating β‐catenin signals. Finally, the LMS‐based NGCs promoted nerves regeneration and recovered motor functions in a sciatic nerve injury model. Similarly, Ca_7_MgSi_4_O_16_ bioceramic‐based NGCs also promoted functional nerves regeneration through immunomodulation and vascularization.^[^ ^149^ ^]^

Electroactive inorganic materials have been found to promote neurite outgrowth and enhance electrical signals transmission, which inspired researchers to integrate conductive materials into NGCs to remodel the bioelectrical microenvironments. Besides, NGCs with 3D porous topological structures are beneficial to cell adhesion and migration, providing more biomimetic microenvironments. 4D printing is an advanced manufacturing technique that possesses stimuli‐responsive properties to form complex biomimetic structures. In a recent study, conductive Ti_3_C_2_T*_x_* MXenes nanosheets were incorporated into polymers with temperature induced‐shape memory properties for the fabrication of NGCs.^[^ ^150^ ^]^ The printed scaffolds could automatically roll into a conduit‐like structure at 37 °C, enabling to wrap the damaged nerve fibers; thus, greatly decreasing surgical time. Ti_3_C_2_T*_x_* MXenes nanosheets endowed the scaffolds with favorable conductivity, which not only promoted the proliferation, migration, and maturation of SCs but also supported to restore the complete electrical pathway of injured nerves.

In addition, external fields (e.g., electric, ultrasound, magnetic, and light) have also been proved to improve the performance of electroactive materials for regulating neural cell behaviors. Due to exogenous electrical stimulation being an invasive treatment— the implantation of electrode would increase the risk of infection and induce local inflammation—several wireless and self‐powered strategies were developed recently. As an example, a wireless self‐powered NGCs composed of CNTs, GelMA, and PLLA fibers was prepared for nerves regeneration.^[^ ^78a^ ^]^ Body movement would induce the generation of mild electricity of PLLA, and CNTs provided favorable conductive microenvironments; thus, synergistically enhancing the SCs adhesion as well as promoting the axonal outgrowth of neurons. In another study, researchers attempted to incorporate microneedles into NGCs to integrate mechanical force‐coupled electrical stimulation (Figure 4B).^[^ ^84^ ^]^ Morphologically, the outer layer of NGCs is an array of microneedle tips for stimulating the surrounding muscles; while, the inner layer is an aligned channel with favorable conductivity and piezoelectricity composed of ZnO nanoparticles, rGO, and PCL. Under the exercise treatment, the inner layer can generate microcurrent to stimulate SCs migration, myelination, and neurite growth; while, the electric signals are also transmitted into the muscles via outer layer microneedles, both synergistically promoting nerves regeneration and inhibiting muscles atrophy. However, it should be noted that the piezo‐electric signals generated by body movement or passive motion are relatively weak. Ultrasound (US), a newly remote mechanical stimulation source, was regarded as a safe and promising approach for piezoelectric stimulation.^[^ ^151^ ^]^ As an example, US‐triggered multifunctional NGCs with aligned piezoelectric nanofiber inner layer and thermo‐responsive NGF release hydrogel out layer were fabricated for nerve regeneration.^[^ ^152^ ^]^ Inner barium titanate piezoelectric nanoparticles (BTNPs) modified‐P(VDF‐TrFE) aligned nanofibers could provide topographical cues and US‐triggered electrical signals for promoting neurite outgrowth and differentiation. Besides, US stimulation also increased the temperature to trigger the shrinkage of outer hydrogel, which achieved the controllable release of NGF to promote the neuron outgrowth. Finally, these multifunctional NGCs, combined with US stimulation, effectively promoted the nerve regeneration and motor functional recovery in a long sciatic nerve defects model. Moreover, light stimulation is an emerging type of non‐invasive stimulation approach for nerve regeneration.^[^ ^153^ ^]^ Up‐conversion nanoparticles (UCNPs)‐based NGCs enable converting NIR excitation into blue emission, which further activates blue light‐responsive ChR2 ions channels on the surface of SCs; thus, improving its proliferation, migration, and myelination activities.^[^ ^154^ ^]^ This study indicated that photo responsive materials‐based optogenetics strategies represent great potential in neural tissue engineering.

Magnetic stimulation has also been used in peripheral nerves regeneration. As an example, rGO were incorporated into SCs‐laden hydrogel to endow the bioprinted scaffolds with electrophysiological properties.^[^ ^155^ ^]^ Under the pulsed electro–magnetic field stimulation, the scaffolds promoted the adhesion, neurogenesis‐related genes, and S‐100β protein expression of encapsulated SCs. Extracellular vesicles (EVs) contain abundant bioactive molecules such as RNA, cytokines, and lipids, which are attracting increasing attention in regenerative medicine.^[^ ^156^ ^]^ Besides, the production and component of EVs can be regulated by physical/chemical stimulation. In a study, the authors designed a composite hydrogel composed of polyethyleneglycol/polyethyleneimine coated superparamagnetic nanoparticles (PEG/PEI‐SPIONs), polyacrylamide/hyaluronic acid (PAAm/HA), and SCs for the on‐demand production and secretion of SCs‐derived EVs (Figure 4C).^[^ ^46^ ^]^ Under the rotating magnetic field, magnetic nanoparticles could act as massages that generate mechanical force on encapsulated SCs to greatly increase the production of EVs via activating Piezo2 pathway. Interestingly, the released EVs possessed multiple bioactivities, including alleviating inflammation, angiogenesis, and axon outgrowth, thereby contributing to nerve regeneration and functional recovery.

### 4.3 Bone

**Figure 5:**
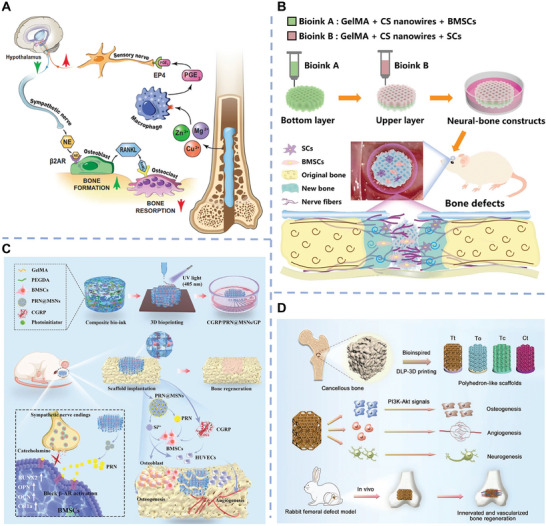
Inorganic biomaterials used for innervated bone regeneration. A) Divalent cations including Cu, Mg, and Zn ions could induce macrophage secreting PGE2, which then bound with EP4 receptor of sensory nerves to inhibit sympathetic nerves activity; thus, leading to enhanced osteogenesis. Reproduced with permission.^[^ ^159^ ^]^ Copyright 2022, Springer Nature. B) Schematic illustration of 3D bioprinted calcium silicate bioinks‐based multicellular constructs for innervation bone regeneration. Calcium silicate nanowires were acting as bioactive agents to promote the osteogenic differentiation of BMSCs and neurogenic differentiation of SCs; thus, leading to bone regeneration and innervation. Reproduced with permission.^[^ ^123^ ^]^ Copyright 2022, Elsevier. C) Schematic diagram of bioprinted constructs for enhanced bone regeneration through inhibiting sympathetic nerves activity and enhancing sensory nerves activity. Reproduced with permission.^[^ ^173^ ^]^ Copyright 2023, Wiley‐VCH GmbH. D) Schematic illustration of polyhedron‐like bioceramic scaffolds for innervated and vascularized bone regeneration. Due to its superior spatial topological properties, polyhedron‐like scaffolds enabled promoting osteogenesis via PI3K‐AKT signal pathway, angiogenesis, and neurogenesis. Reproduced with permission.^[^ ^109^ ^]^ Copyright 2023, Wiley‐VCH GmbH.

In the past few decades, increasing evidence has confirmed the crucial role of the nervous system on bone development, metabolism, and regeneration.^[^ ^157^ ^]^ Nerves are densely distributed in the bone, including periosteum, cortical bone, cancellous bone, and bone marrow.^[^ ^6^ ^]^ Sensory and sympathetic nerves can secrete numerous neuropeptides and neurotrophic factors that act on bone‐resident cells (BMSCs, endothelial, cells and macrophages) to modulate regenerative microenvironments, thereby regulating bone regeneration.^[^ ^158^ ^]^ Owing to the easily controllable chemical composition and physical micro/nano structure, inorganic biomaterials have attracted more attention in bone regeneration with innervation.^[^ ^7^ ^]^ Interestingly, recent studies also confirmed that the promotion of osteogenesis by bioactive metal ions is mediated by nervous system (**Figure** **5**A).^[^ ^159^ ^]^ As an example, Zhang et al. found that the released Mg ions from the implant could act on DRG neurons to induce the secretion of CGRP, which further promoted the osteogenic differentiation of stem cells.^[^ ^19^ ^]^ Further study demonstrated that CGRP induced by Mg‐based implants could also activate the CGRP‐FAK‐VEGF pathway to induce angiogenesis.^[^ ^160^ ^]^ These studies highlighted the multifunctional activities of Mg ions, especially in neurogenesis. In another study, a bilayer hydrogel containing Mg‐incorporated BP nanosheet was developed for neuro‐vascularized bone regeneration.^[^ ^67^ ^]^ The Mg‐BP sheet effectively stimulated the neuronal differentiation of NSCs and angiogenic activity of HUVECs. In vivo results showed that the composite hydrogel promoted early innervation and vascularization, which contributed to enhanced bone regeneration. Apart from Mg ions, SiO_3_^2−^ ions have suitable neurotrophic effects that could enhance the metabolic activity of neurons.^[^ ^161^ ^]^ In a study, silicified collagen scaffolds were prepared for critical bone regeneration.^[^ ^24^ ^]^ Silicon ions induced the axon outgrowth and secretion of Sema3A of DRG through activating PI3K‐Akt‐mTOR pathway, that in turn, promoted angiogenesis and osteogenesis. Recently, researchers have tried introducing inorganic biomaterials into bioinks to enhance its bioactivities. The released multiple ions could create a beneficial ionic microenvironment to promote the survival, proliferation, and specific differentiation activities of encapsulated cells. As an example, calcium silicate (CS) nanowires were incorporated into GelMA hydrogel; and then, combined with BMSCs and SCs to prepare multicellular constructs (Figure 5B).^[^ ^123^ ^]^ Bioactive Ca and Si ions released from constructs not only supported the long‐term survival and spreading of cells but also promoted the osteogenic differentiation of BMSCs and neurogenic differentiation of SCs. The multicellular constructs containing CS nanowires promoted new bone formation and osteointegration and induced nerve fibers ingrowth, achieving innervated bone regeneration. Neural stem cells can differentiate into neurons and secret neuropeptides, which is helpful for innervation.^[^ ^162^ ^]^ In a recent study, a neural construct composed of lithium calcium silicate (LCS) bioceramic and neural stem cells (NSCs) was proposed for innervated bone regeneration.^[^ ^140^ ^]^ Bioactive ions released from LCS bioceramic promoted the proliferation and neuronal differentiation of NSCs within the constructs via the activation of PI3K‐AKT pathway. Besides, the expression level of CGRP was also upregulated, which further stimulated angiogenesis and osteogenesis via the paracrine route. Consequently, remarkable bone regeneration with vascularization and innervation was observed after the implantation of neural constructs into bone defects.

Due to the sensitivity of both bone and nerves to electrical signals, inorganic electroactive materials such as MXenes, carbon‐based materials, are regarded as promising therapeutic agents to promote innervated bone regeneration. As an example, an injectable hydrogel composed of Cu‐modified germanium phosphorus nanosheet and GelMA matrix was designed for innervated and vascularized bone repair.^[^ ^68^ ^]^ The conductive hydrogel promoted the neurite growth of PC12 cells and neuronal differentiation of NSCs, and the released Cu^2+^ ions also enhanced the angiogenic activity of HUVECs, thereby leading to neuro‐vascularized bone regeneration. In another study. Mo_2_Ti_2_C_3_ nanosheets were incorporated into gelatin and deacetylated chitosan to form composite hydrogel for bone regeneration.^[^ ^163^ ^]^ The in vivo results showed that MXenes hydrogel obviously promoted new bone formation and induced host nerves ingrowth. Moreover, rGO was combined with hydrogel for the fabrication of 3D printed hybrid scaffolds. The incorporation of rGO endowed scaffolds with excellent osteogenic and neurogenic activities; thus, greatly inducing osteogenesis and neurogenesis after subcutaneous implantation.^[^ ^164^ ^]^ In addition, piezoelectric materials could generate electrical charges under mechanical force, which is similar to bone tissues that possess piezoelectricity. Whitlockite (WH) is a natural mineral containing Ca, Mg, and P elements, which also possesses suitable piezoelectricity.^[^ ^165^ ^]^ 3D printed composite scaffolds composed of WH and PCL were used for bone regeneration.^[^ ^166^ ^]^ The piezoelectric property and sustained release of Mg ions synergistically enhanced the neurogenic, angiogenic, and osteogenic differentiation activities in vitro. 3D printed composite scaffolds effectively promoted neuro‐vascularized bone regeneration in the critical calvarial defect model. Electret is a dielectric material that can quasi‐permanently store electrostatic charges to provide an external electrostatic field, including SiO_2_, ZnO, and BaTiO_3_. In a recent study, an aligned core–shell composite membrane of PCL/SiO_2_ in core layer and PLLA in shell layer was prepared by coaxial electrospinning.^[^ ^167^ ^]^ The composite membrane, combined with electrical stimulation, promoted the myelination of Schwann cells via inhibiting the Notch pathway, and modulated macrophage polarized toward M2 phenotype. The composite membrane effectively promoted innervated bone regeneration after implantation into the defects for 8 weeks.

In addition, inorganic biomaterials are also utilized to deliver neurotransmitters to regulate neural activity for bone regeneration. It is already known that NGF‐TrkA signals are essential for bone development and regeneration.^[^ ^168^ ^]^ Laponite, a disk‐shaped 2D silicate material, was served as carrier to load NGF, and then, incorporated into the GelMA/AlgMA hydrogel for the fabrication of bone constructs.^[^ ^169^ ^]^ The slow release of NGF from the constructs could mimic the ossification center microenvironment, that induced the axon outgrowth and CGRP secretion of sensory neurons, thereby promoting osteogenesis through activating AMPK‐PPAR signals. In vivo results showed that the implantation of constructs obviously increased the neural and vascular networks density. Moreover, post‐traumatic stress disorder usually induced the over‐activation of sympathetic nerves to secret excessive catecholamine, which had negative effects on bone repair.^[^ ^170^ ^]^ The released norepinephrine could bind with 𝛽‐adrenergic receptors (𝛽‐AR) expressed on the surface of osteoblast, thereby inhibiting bone formation.^[^ ^171^ ^]^ Propranolol (PRN), a non‐selective 𝛽‐adrenergic receptor blocker, has been demonstrated to inhibit sympathetic nerves activity.^[^ ^172^ ^]^ As an example, composite bioinks containing mesoporous silica nanoparticles (MSNs)‐loaded with PRN, CGRP, BMSCs, GelMA, and PEGDA were used for 3D bioprinting of scaffolds with neuro‐modulatory microenvironments (Figure 5C).^[^ ^173^ ^]^ The scaffolds could enhance the sensory nerves activity due to the release of CGRP; while, inhibiting sympathetic nerves activity due to the release of PRN, which synergistically promoted the osteogenic differentiation of BMSCs and contributed to bone regeneration in vivo.

Apart from chemical composition, micro/nano topographical structures of inorganic biomaterials also possessed remarkable effects on the behaviors of both nerves and bone. The effects of stiffness, roughness, and porosity characteristics of biomaterials on osteogenesis and neurogenesis can be found in previous review articles. As an example, inspired by the phyllotaxis of plant, Zhang et al. designed and prepared tree‐like bioceramic scaffolds to deliver BMSCs and SCs for inducing innervated bone regeneration.^[^ ^32^ ^]^ The blade interval and divergence angles of scaffolds could be easily modulated through programmed design. More importantly, the leaf blades supported the spatial distribution of multiple cells, and its surface microstructure effectively enhanced the neurogenic activity of SCs and osteogenic activity of BMSCs, thereby promoting innervated bone regeneration. In another study, space‐filling polyhedron‐like bioceramic scaffolds were designed to mimic the complex meshwork structure of cancellous bone.^[^ ^109^ ^]^ As compared with the traditional scaffolds with cross stacked structure, polyhedron‐like scaffolds provided more suitable average static pressures to support cell adhesion. Besides, the spatial structures of scaffolds possessed favorable osteogenesis, angiogenesis, and neurogenesis properties via the activation of PI3K‐AKT pathway. In vivo results showed that polyhedron‐like scaffolds obviously promoted innervated and vascularized bone regeneration, without incorporating additional cells and cytokines, highlighting the great significance of structural design (Figure 5D).

### 4.4 Dental Tissue

**Figure 6:**
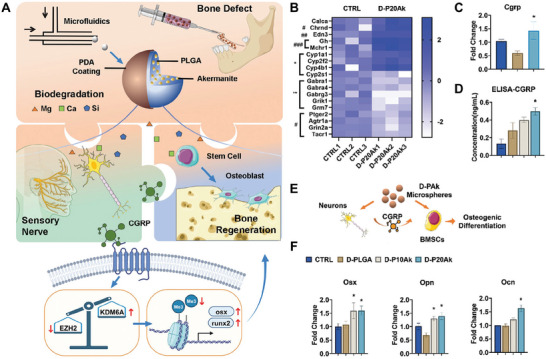
A) Schematic illustration of injectable Ca–Mg–Si bioceramic‐based microspheres for neural‐mediated maxillofacial bone regeneration. B) Heat‐map of the different expressed genes of CTRL and D‐P20Ak groups. C) CGRP genes expression. D) ELISA test of CGRP secretion. E) The interaction of D‐PAK microspheres, neurons, and BMSCs. F) The osteogenic‐related genes expression of osteoblast in different groups. Reproduced with permission.^[^ ^179^ ^]^ Copyright 2024, Wiley‐VCH GmbH.

As another part of the mineralized component of human body, inorganic‐based biomaterials have been widely used in dental tissues application.^[^ ^174^ ^]^ Many researchers have studied the potential of inorganic‐based biomaterials for the treatment of periodontitis, pulp regeneration, maxillofacial defects, and others.^[^ ^175^ ^]^ For example, periodontitis is a chronic and infectious oral disease caused by dental plaque, and the accumulation of ROS and pro‐inflammatory cytokines such as interleukin‐1 beta (IL‐1β) and tumor necrosis factor alpha (TNF‐α) leads to the outcome of tooth loss.^[^ ^176^ ^]^ Hence, remodeling immune microenvironments and enhancing the osteogenesis activities are regarded as promising options. For instance, Ming et al. developed biomimetic microspheres containing sericin‐hydroxyapatite nanoparticles (Se‐nHA) and proanthocyanidins (PC) for alleviating alveolar bone loss in periodontitis.^[^ ^176b^ ^]^ The in vitro results showed that PC effectively scavenged ROS and induced transformation of macrophage from M1 toward M2 phenotype; while, Se‐nHA obviously stimulated the osteogenic differentiation of stem cells. The microspheres improved the alveolar bone regeneration in a rat periodontitis model. Histological analysis revealed that nerve fibers densely distribute in alveolar bone and dental pulp, which plays an important role in transmitting the sense of pain.^[^ ^177^ ^]^ Moreover, nerves also participate in modulating inflammatory response and angiogenesis through secreting neurotrophic factors and neuropeptides. Re‐innervation is indispensable for the restoration of sensation functions of regenerated dental pulp. In one study, conductive hydrogel coupled with endogenous electric fields was developed to enhance the osseo‐integration and osseo‐perception of dental implants.^[^ ^178^ ^]^ This hydrogel improved the axonal outgrowth and neuropeptides secretion behaviors of neurons through increasing intracellular Ca^2+^ concentration, and subsequently, activating PI3K‐AKT and MEK‐ERK signal pathways. Besides, conductive hydrogel also indirectly promoted the osteogenic differentiation of osteoblast via neuro‐modulation effects. The in vivo results showed that conductive hydrogel effectively induced sensory nerves innervation, which not only promoted the osteointegration of dental implant with surrounding bone but also enhanced the sensitivity to electric and temperature stimuli. Moreover, inorganic‐based biomaterials have also been used in maxillofacial bone defects. To better fit the irregular morphology of defect site, an injectable hydrogel containing PDA‐PLGA‐AKT microspheres was fabricated via microfluidics.^[^ ^179^ ^]^ AKT bioceramic had the sustained release profile of Ca, Mg, and SiO_3_^2−^ ions, which is already acknowledged as osteogenesis and neurogenesis ability. The results showed that bioactive ions could upregulate the secretion of CGRP from trigeminal neurons, subsequently the released CGRP inhibited EZH2 expression and promoted KDM6A expression of BMSCs to initiate the osteogenic differentiation process (**Figure** **6**). Generally, constructing inorganic based‐biomaterials with neuroactive properties has great potential for functional pulp regeneration.

### 4.5 Skeletal Muscles

Skeletal muscle is the most abundant tissue and accounts for ≈40% weight of human body.^[^ ^180^ ^]^ Skeletal muscles injury caused by trauma, orthopedic surgeries, and degenerative disease would significantly affect the life quality of patient due to the skeletal muscles directing the dynamic movement of human body.^[^ ^181^ ^]^ Skeletal muscles have remarkable regenerative ability following minor damages; while, the volumetric muscle loss (VML) can overwhelm its inherent regenerative capabilities and lead to permanent functional impairment.^[^ ^182^ ^]^ As a highly innervated tissue, the contraction of myofibers is controlled by somatic motor neurons through neuromuscular junctions (NMJs). It is reported that denervated muscles would lose contraction capability; thus, gradually undergoing degeneration and atrophy.^[^ ^183^ ^]^ Hence, it is urgently required to develop novel strategies of delivering cells and growth factors, or provide biophysical and biochemical cues of biomaterials to repair the damaged muscles and host nerves integration.^[^ ^184^ ^]^

Recently, 3D bioprinting technology has emerged as an effective approach to recapitulate the physiological structure of native skeletal muscles.^[^ ^120^ ^]^ As an example, 3D bioprinted muscle constructs with aligned topological structures were prepared for the treatment of VML.^[^ ^185^ ^]^ The physical cues of filament promoted the myogenic differentiation of human muscle progenitor cells and myofibers formation; thus, contributing to muscle regeneration with accelerated innervation. Moreover, due to the electrical sensitivity of muscles and nerves, electrical stimulation, electroactive biomaterials, or both combinations hold great potential in promoting innervated muscle regeneration. For example, Kim et al. developed a bioengineered muscle construct via in situ electrical stimulation‐supplemented 3D bioprinting technique.^[^ ^186^ ^]^ In situ electrical stimulation significantly activated the voltage‐gated ion channels to induce the cytoskeleton alignment and myogenic differentiation of loaded stem cells. The bioprinted constructs promoted muscles regeneration and functions recovery after implanting into the VML defects. In another study, Kang et al. prepared GO‐containing bioinks to fabricate myoblast‐laden scaffolds.^[^ ^187^ ^]^ The results showed that electroactive bioinks can provide suitable microenvironment to induce myogenic differentiation of myoblast. Besides, the combination of electric stimulation and electroactive biomaterials may play synergistic effects on regulating cell behaviors. For example, electroactive Au nanowires containing‐bioinks were introduced and combined with electric field‐reinforced bioprinting process to prepare biomimetic cell‐laden scaffolds.^[^ ^128^ ^]^ On the one hand, the electric field can provide anisotropic electrical microenvironments to mimic the electrical properties of native muscle tissues. On the other hand, the external electric field can promote the orientation of Au nanowires, which further provide topographical cues to induce myoblast alignment and myogenic differentiation. Both synergistically promote innervated muscle regeneration.

**Figure 7:**
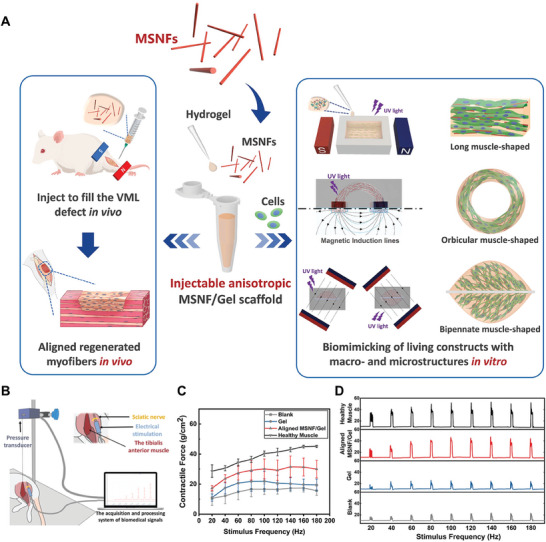
A) Schematic illustration of injectable PCL‐CNT‐Fe3O4 magnetic nanofibers (MSNFs)‐based hydrogel for skeletal muscles regeneration. Aligned magnetic nanofibers under magnetic field could promote cell alignment in vitro and induce myofibers formation in vitro. B) Diagram of the measurement of contractile force of regenerated muscles. C) Contractile force at different frequency. D) Representative traces of muscles. Reproduced with permission.^[^ ^188^ ^]^ Copyright 2022, Elsevier.

Apart from 3D bioprinting, hydrogel containing inorganic biomaterials is also a promising approach for muscle regeneration. As an example, injectable hydrogel scaffolds containing aligned PCL‐CNT‐Fe_3_O_4_ magnetic nanofibers were prepared for anisotropic skeletal muscle regeneration.^[^ ^188^ ^]^ Magnetic nanofibers were well oriented under the magnetic field, which could provide 3D micro/macro‐structure cues to guiding myoblast alignment and myofibers formation (**Figure** **7**). A VML model of rat was established to demonstrate that this hydrogel scaffold significantly accelerated skeletal muscles regeneration and largely restored its contractile functions. Moreover, recent studies found that bioactive ions also have positive effects on muscle regeneration. For example, Xu et al. developed a multifunctional hydrogel containing SiO_3_^2−^ ions for enhanced muscle repair.^[^ ^189^ ^]^ The sustained release of SiO_3_^2−^ ions can create a suitable ionic microenvironment to promote the proliferation and myogenic differentiation of C2C12 cells, inducing angiogenesis. In another study, Yuan et al. developed an injectable hydrogel composed of alginate, strontium carbonate, and calcium silicate particles.^[^ ^190^ ^]^ The released Sr and SiO_3_^2−^ ions could synergistically promote the expression of muscle‐regulating factors (e.g., MyoG, MyoD) of C2C12 cells and induce M2 macrophage polarization to inhibit muscle necrosis.

### 4.6 Tendon

**Figure 8:**
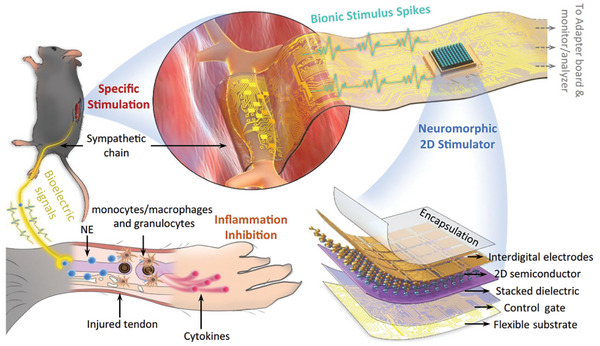
Schematic illustration of neuromorphic electro‐stimulation for inhibiting tendon inflammation via neural‐regulation pathway. Bioelectric signals generated from the implanted neuromorphic electro‐stimulation could enhance NE release; thus, inhibiting expression of inflammatory cytokines. Reproduced with permission.^[^ ^195^ ^]^ Copyright 2024, Springer Nature.

The tendon is an integral part of the musculoskeletal system that is mainly responsible for transmitting force from muscle to bone.^[^ ^191^ ^]^ Different from other tissues, the tendon has low degree of innervation; so, peripheral nerves only distribute on the surface of tendon (paratenon, endotenon, and epitenon) instead of entering the tendon proper.^[^ ^192^ ^]^ Interestingly, it is reported that sensory nerves can sprout and grow into tendon proper after injury, and then, retract to the surface after the healing process is finished.^[^ ^193^ ^]^ Specifically, the ingrowth of sensory nerves promotes tendon sheath progenitor cells proliferation and vascularization to repair injured tendon. Another study reported that in situ injection of substance P induced the ingrowth of endogenous sensory nerves, thereby contributing to tendon repair.^[^ ^194^ ^]^ Further, the nervous system also regulates the acute inflammatory response after tendon injury. In a recent study, an atomically thin MoS_2_‐based neuromorphic electric stimulator was proposed for the treatment of tendon injury (**Figure** **8**).^[^ ^195^ ^]^ After being wrapped in the surrounding sympathetic chain that innervated tendon segment, the stimulator promoted the release of noradrenaline of neurons, which further suppressed the expression of inflammation‐associated cytokines IL‐6 to alleviate the acute inflammation response.

### 4.7 Skin

As the first barrier to defend the body from external invasion, the skin is easy to be destroyed by accident, burns, and diseases.^[^ ^196^ ^]^ Currently, the treatment of skin wounds aims to not only promote wound healing but also restore its physiological functions. Skin is densely innervated by nerve fibers, which discriminate pain, temperature, and perception.^[^ ^197^ ^]^ Therefore, re‐innervation is indispensable for functional skin regeneration, but remains challenging.^[^ ^198^ ^]^

**Figure 9:**
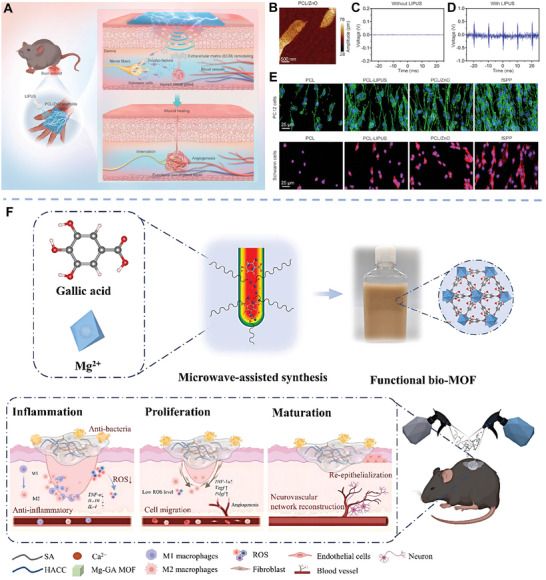
Inorganic biomaterials used for innervated skin regeneration. A) Schematic illustration of flexible sono‐piezo patch for functional wound healing. The patch composed of PCL and ZnO nanoparticles enabled building of an endogenous pro‐regenerative microenvironment under LIPUS that effectively promoted angiogenesis and neurogenesis, thereby promoting functional wound healing. B) PFM amplitude mapping of the composite patch. C,D) Output voltage of the patch with or without LIPUS stimulation. E) PCL/ZnO composite patch combined with LIPUS obviously promoted the neurite outgrowth of PC12 cells and neurogenic differentiation of Schwann cells. Reproduced with permission.^[^ ^89^ ^]^ Copyright 2024, American Chemical Society. F) Schematic diagram of Mg‐GA MOFs‐based sprayable hydrogel for neuro‐vascularized skin regeneration of diabetic wounds. The released GA could scavenge ROS and modulate macrophages polarized toward M2 phenotype; while, Mg ions effectively induced angiogenesis and neurogenesis. Reproduced with permission.^[^ ^206^ ^]^ Copyright 2024, Elsevier B.V.

Similar to other tissues, the skin is also sensitive to electrical signals, and previous studies have demonstrated that electrical stimulation could promote wound healing and innervation.^[^ ^199^ ^]^ However, the usage of external power devices and wires may cause many inconveniences in clinical. To solve this problem, Tan et al. designed a two‐layered self‐powered smart patch; the PVDF film layer served as piezoelectric generator to provide electric signals and the GelMA hydrogel containing rGO, cytokines, and exosomes acted as conductor layer to transmit electrical and biological cues.^[^ ^200^ ^]^ The generated electric signals could induce the neurogenic differentiation of BMSCs through activating PI3K‐AKT pathway. The smart patches effectively accelerated cutaneous innervation and sensation functions restoration. Moreover, ultrasound, light, and magnetic driven‐wireless stimulation strategies are also feasible options. In a recent study, a flexible sono‐piezo composed of PCL and ZnO nanoparticles was prepared as wound dressing (**Figure** **9**A–E).^[^ ^89^ ^]^ ZnO nanoparticles possess effective piezoelectric activities, which can generate hundreds of mV of output voltages with ultrasound excitation. PCL/ZnO scaffolds, combined with ultrasound excitation, obviously promote neurite outgrowth of PC12 cells and neurotrophic factors‐related genes expression of SCs, which further induce angiogenesis and functional maturation of sweat gland organoids. More importantly, the in vivo results showed that this sono‐piezo modulation strategy effectively accelerated wound healing, sensory function recovery, and functional sweat glands restoration. Moreover, a collagen/hyaluronic acid hydrogel containing red light‐responsive TiO_2_/Bi_2_S_3_ nanotubes was prepared for the treatment of deep burns wound healing.^[^ ^201^ ^]^ Electrical signals generated by TiO_2_/Bi_2_S_3_ nanotubes under the excitation of red light could stimulate the neuronal differentiation and metabolic activity of PC12 cells in vitro and promote innervation in vivo. Interestingly, skin injury can spontaneously trigger the formation of endogenous electric fields, which inspired us that developing conductive biomaterials‐loaded wound dressing might also beneficial to innervation. As an example, Fan et al. fabricated a hydrogel wound dressing containing polypyrrole conductive nanoparticles for diabetic wound healing.^[^ ^202^ ^]^ Conductive hydrogel effectively promoted neurogenesis and angiogenesis through the activation of PI3K/AKT and MEK/ERK pathways.

Recently, bioactive ions (e.g., Mg, Zn, and Cu) have been confirmed to promote cutaneous innervation. For example, Zhang et al. developed fibrous scaffolds containing Zn_2_SiO_4_ nanoparticles for the repair of deep burn wounds.^[^ ^88^ ^]^ The scaffolds could sustain release of bioactive Zn and SiO_3_^2−^ ions to stimulate neurogenesis and angiogenesis, thereby promoting innervated wound healing. Another study reported magnesium silicate containing sprayable hydrogels for wound healing and appendages regeneration.^[^ ^203^ ^]^ The released Mg and SiO_3_^2−^ ions promoted angiogenesis and neurogenesis and modulated inflammatory response. The in vivo results showed that sprayable hydrogels effectively promoted wound healing with innervation, vascularization, and hair follicles regeneration, as compared with commercial products Dermlin and 45S5 bioglass. Besides, Cu and Mg‐doped bioactive glass were incorporated into hydrogels to promote infected wound healing because Cu ions had photothermal antibacterial and angiogenesis ability and Mg ions could enhance neurogenesis.^[^ ^204^ ^]^ In another example, Zhang et al. designed a composite wound dressing composed of Cu‐doped calcium silicate powers and curcumin, utilized for deep burn healing.^[^ ^205^ ^]^ The released Cu ions and curcumin could spontaneously form chelates to promote hair follicle regeneration, which accelerated the expression of nerve‐related factors (e.g., β‐Tubulin and GFAP) and contributed to innervation.

MOFs also attracted more attention in wound healing and innervation due to their high biocompatibility and large surface area. As an example, a sprayable hydrogel containing magnesium‐gallate (Mg‐GA) MOF was applied for diabetic wound healing (Figure 9F).^[^ ^206^ ^]^ The released GA could scavenge ROS and suppress inflammation; while, Mg ions contributed to enhance angiogenesis and neurogenesis. After implantation into the defect areas, the hydrogel accelerated wound healing with vascularization and innervation. Besides, MOF can also serve as carriers to in situ delivering drugs and proteins. For example, vancomycin was loaded into Zn–curcumin MOF, and then, incorporated into GelMA/oxidized alginate hydrogels for infected wound healing.^[^ ^207^ ^]^ The released vancomycin and Zn ions synergistically killed bacteria, and curcumin could modulate the polarization of macrophage, thereby promoting innervated and vascularized skin regeneration. In another study, Zn‐pyridine‐2,6‐dicarboxylic acid (NU‐801) MOF loading with intracellular proteins and coated with neuroblastoma membrane, was prepared for the burn wound healing.^[^ ^208^ ^]^ The external cell membrane coating decreased immune rejection and inflammation, and internal loaded proteins contributed to neurogenesis and hair follicle regeneration. The in vivo results showed the effectiveness of these materials for functional skin regeneration.

### 4.8 Corneal Tissue

Nerves fibers are densely distributed in corneal tissues that originate from the ophthalmic branch of trigeminal nerves and radiate into the cornea from the periphery.^[^ ^209^ ^]^ As a part of corneal tissues, corneal nerves play an important role in controlling blink reflex, regulating tear production and secretion. Besides, corneal nerves also maintain the physiological homeostasis through secreting numerous neurotrophic factors.^[^ ^210^ ^]^ Recently, inorganic biomaterials have been applied to corneal regeneration and corneal nerves repair. As an example, Zheng et al. developed a stimulation electrode composed of polyaniline functionalized graphene (PAG) for stimulating corneal nerves regeneration.^[^ ^209^ ^]^ With the electrical field, the stimulation electrode could activate Ca ions‐related MAPK signaling pathway to promote the axons outgrowth of trigeminal ganglion neurons; thus, increasing the corneal nerves density in vivo. In another work, aligned nanofibrous scaffolds containing poly‐pyrrole functionalized‐graphene were applied as stimulation electrode for the treatment of glaucoma.^[^ ^211^ ^]^ The aligned nanofibrous scaffolds with electrical stimulation significantly enhanced the survival, neurite outgrowth, and anti‐aging abilities of retinal ganglion cells, which provides a new approach for the treatment of optic nerves injury diseases.

**Figure 10:**
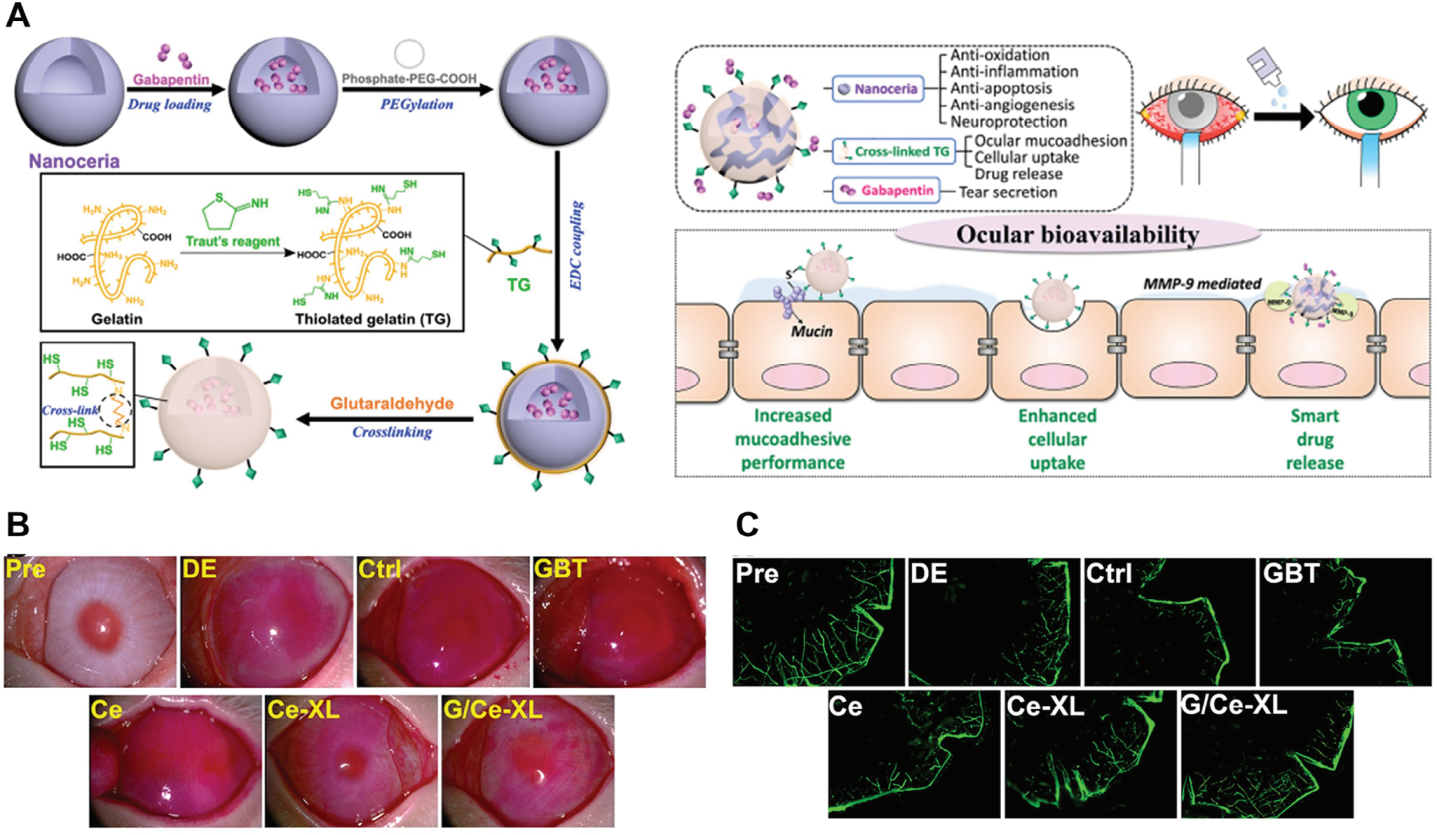
A) Schematic illustration of CeO_2_‐based nanoplatform for dry eye treatment. B,C) CeO_2_‐based nanoplatform exhibiting superior cellular protective and neuronal protective activities in vivo. Reproduced with permission.^[^ ^213^ ^]^ Copyright 2023, American Chemical Society.

In addition, inorganic biomaterials can also serve as an ideal drug delivery platform for ocular condition treatment, such as keratitis, uveitis, and dry eyes. As an example, Yu et al. developed a smart nanoplatform containing UCNPs@Bi@SiO_2_ nanoparticles with genistein loaded.^[^ ^212^ ^]^ The nanoplatform possessed suitable biocompatibility and NIR‐triggered release profile of genistein, exhibiting promising potential for the treatment of angiogenesis‐related ocular diseases. Moreover, cerium oxide (CeO_2_) nanoparticles coated with thiolated gelatin, followed by crosslinking with gabapentin, were developed for dry eye treatment (**Figure** **10**).^[^ ^213^ ^]^ The CeO_2_ nanoparticles showed remarkable anti‐oxidation, anti‐inflammation, anti‐apoptosis, and neuronal protective properties; while, the released gabapentin contributed to tear secretion. The nanoplatform was utilized to repair corneal epithelial injuries and maintain the corneal nerves density in the rabbit model of dry eye disease.

### 4.9 Cardiac Tissue

**Figure 11:**
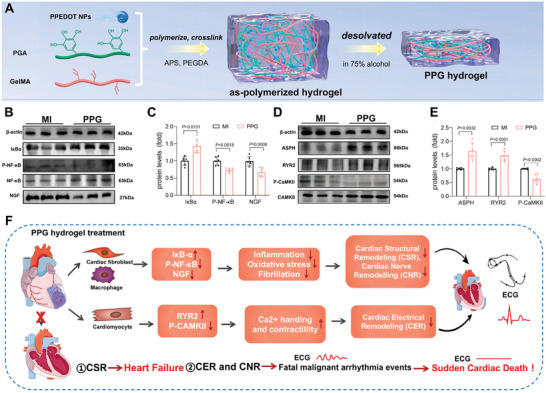
A) Schematic illustration of the design and preparation of conductive hydrogel patch. B,C) Protein expression and statistical analysis of NF‐κB signal pathway. D,E) Protein expression and statistical analysis of CaMKII‐RyR2 signal pathway. F) Diagram of mechanism of conductive hydrogel patch on remodeling myocardial infarction. Reproduced with permission.^[^ ^219^ ^]^ Copyright 2024, Elsevier.

Myocardial infarction is a major cause of death worldwide, which is induced by acute and persistent myocardial ischemia and hypoxia due to coronary artery occlusion.^[^ ^214^ ^]^ Due to the inability of adult myocardial cells to proliferate, a novel therapeutic intervention needs to be developed for cardiac repair.^[^ ^215^ ^]^ Recently, biomaterial‐based strategies have been regarded as promising for cardiac tissue regeneration through modulating the immune microenvironment, inducing angiogenesis and enhancing myocardial cells functions.^[^ ^216^ ^]^ However, to date, the crucial roles of the nervous system in cardiac repair have not attracted enough attention. The heart is densely innervated by sympathetic and parasympathetic nerves that are coordinately responsible for maintaining its physiological functions, such as rhythm, contraction forces, and output of cardiac.^[^ ^217^ ^]^ It is reported that cardiac denervation or disordered neuronal activities would induce arrhythmia and impair cardiac regeneration ability.^[^ ^218^ ^]^ Besides, a recent study showed that aging significantly decreased the nerves density of heart, leading to ventricular tachycardia; while, anti‐aging drugs treatment could restore nerve density.^[^ ^5b^ ^]^ In a recent study, Chen et al. designed a conductive and wet‐adhesive hydrogel patch composed of poly gallic acid, GelMA, and polydopamine‐hybridized PEDOT nanoparticles for repairing myocardial infarction (**Figure** **11**).^[^ ^219^ ^]^ On the one hand, the hydrogel patch reduced the secretion of NGF from macrophages via inhibiting NF‐κB signals, thereby suppressing cardiac structural remodeling and cardiac nerve remodeling to avoid sudden cardiac death. On the other hand, the hydrogel patch activated Ca ions signal transduction to improve cardiac functions. Another work reported that the injection of n‐isopropyl acrylamide coated‐Fe_3_O_4_ nanoparticles could modulate the volume and density of neurons to inhibit atrial fibrillation. Generally, a strategy from the perspective of neural regulation of designing biomaterial‐based therapies will provide more ideas for the treatment of heart‐associated diseases.

### 4.10 Cavernous Tissue

**Figure 12:**
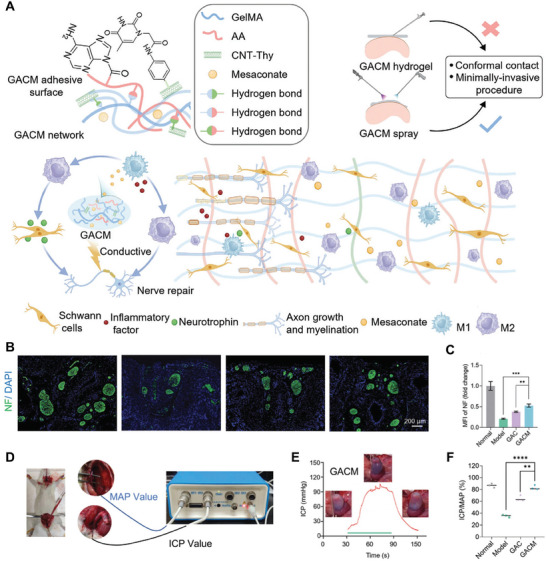
A) Schematic illustration of CNT‐based sprayable hydrogel for repairing cavernous nerves and recovering its functions. The hydrogel could inhibit inflammatory cytokines release via polarized macrophages into M2 phenotypes and promote Schwann cells migration and myelination. B,C) Representative immunofluorescence staining images and statistical analysis of NF in the regenerated cavernous tissues. D) Diagram of the measurement of ICP and MAP values. E,F) Representative results of ICP values and ICP/MAP ratio; these results showed that the erectile functions of cavernous were recovered after treating with sprayable hydrogels. Reproduced with permission.^[^ ^223^ ^]^ Copyright 2024, Wiley‐VCH GmbH.

As the most important component of the penis, cavernous tissues are densely innervated with peripheral nerves that maintain the erectile function and urinary function of the penis.^[^ ^220^ ^]^ However, cavernous nerves injury caused by trauma, surgery, and diseases would result in erectile dysfunction and significantly affect the life quality of men.^[^ ^221^ ^]^ Due to the sensitivity of nerves to electrical signals, inorganic electroactive biomaterials have been emerging as promising options for cavernous nerves repair.^[^ ^222^ ^]^ In a recent study, a sprayable adhesive conductive hydrogel containing gelatin, adenine, carbon nanotubes, and mesaconate was developed for regenerating cavernous nerves and restoring its erectile functions (**Figure** **12**).^[^ ^223^ ^]^ CNTs were grafted with thymine to improve its dispersibility; thus, enhancing the conductivity and mechanical performance of hydrogels. Owing to the suitable electroactivity of CNTs, the conductive hydrogel promoted the neuronal differentiation of neural stem cells and induced the axon outgrowth of DRG neurons. Besides, the conductive hydrogel also promoted macrophage polarized toward M2 phenotype, thereby enhancing the proliferation and neurotrophic factors expression activity of SCs. The in vivo results showed that conductive hydrogel obviously decreased inflammatory response, and promoted the regeneration of myelin sheath and axons. More importantly, the implantation of conductive hydrogel recovered the erectile functions of a damaged penis and restored the fertility. Further, the combination of electroactive materials with external stimulation modes also represents a promising option. A band aid‐like nanopatch composed of aligned BTO‐loaded PCL membranes and GO‐loaded GelMA hydrogel was prepared for repairing cavernous nerves.^[^ ^224^ ^]^ Micro‐currents were stably generated under the stimulation of LIPUS, which effectively stimulated the proliferation and differentiation activity of SCs and promoted axonal growth and myelination of nerves in vivo. Moreover, inorganic biomaterials can also act as carriers to deliver cytokines or drugs such as SDF‐1α, which has good cell recruitment ability. For example, Fu et al. designed an injectable BP@SDF‐1α delivery system for repairing cavernous nerves.^[^ ^225^ ^]^ The sustained release of SDF‐1α recruited the endogenous stem cells into corpus cavernosum and main pelvic ganglion; thus, effectively repairing cavernous nerves and ameliorating erectile dysfunction in rats.

## 5 Conclusions and Perspectives

This review presents the types and properties of the most studied inorganic biomaterials in the fields of innervated multi‐tissue regeneration and inorganic‐based material composites including inorganic biomaterial‐containing membranes, inorganic biomaterial‐containing hydrogel, 3D printing of inorganic biomaterial‐based scaffolds, and 3D bioprinting of inorganic biomaterial‐based cellular constructs. Further, we offer a comprehensive summary of the application of inorganic biomaterials in various types of tissue regenerations including nerve tissues (e.g., central nerves and peripheral nerves) and nerve‐innervated tissues (e.g., bone, skin, and skeletal muscle).

Due to its unique characteristics of tunable chemical composition/structures and physicochemical properties, inorganic biomaterials have excellent tissue‐induction activity and neural induction activity, which show great potential for functional tissue regeneration. The biological activity of inorganic biomaterials is mainly based on the following aspects: 1) Chemical cues: inorganic biomaterials such as bioceramic maintain sustained release of multiple bioactive ions, which can create a beneficial ionic microenvironment to regulate multiple biological activities including osteogenesis, angiogenesis, immunomodulatory, neuro‐induction, and neuro‐conduction.^[^ ^11^ ^]^ Besides, inorganic biomaterials can serve as a delivery platform for controllable loading and releasing of biochemical factors including DNA, RNA, growth factors, and drugs, which could not only enhance its loading efficacy but also protect from degradation. Moreover, several inorganic biomaterials can specifically respond to environments (such as pH, ROS, ultrasound, and magnetic field), thereby exerting catalytic reactions to scavenge inflammatory molecules and produce therapeutic substances such as oxygen and hydrogen to build pro‐regenerative microenvironments.^[^ ^228^ ^]^ 2) Physical cues: inorganic biomaterials could be easily endowed with micro/nano topological structures via 3D printing, hydrothermal, and so on. Topological structures could regulate cell adhesion, proliferation, and differentiation via cell–materials interactions. 3) Electrical cues: electroactive inorganic biomaterials including conductive and piezoelectric biomaterials can transmit or generate electric signals to target cells/tissues. It is worth noting that bioelectricity is an integral part of the human body, and endogenous electrical fields extensively exist in many tissue regeneration processes including bone, skin, corneal, and cardiac tissues. Hence, electric signals could mediate intracellular signal transduction to regulate biological process including mineral deposition, wound healing, muscle contraction, and neuronal differentiation. 4) Combined effects: the rational design of inorganic biomaterials could integrate the above‐mentioned cues with each other to synergistically exert its biological effects on regulating cell behaviors including adhesion, differentiation, and cell–cell interactions.

Although inorganic‐based material systems have shown promising potential in functional tissue regeneration, it is still in the fancy stage with many challenges which will encourage future research in the following aspects:

### 5.1 Pay More Attention to Biosafety

Although inorganic biomaterials showed good biocompatibility in in vitro experiments, it is still required to pay more attention to their long‐term biosafety in vivo. Currently, the assessments of in vivo biosafety are still limited to date, mainly focusing on observing the inflammatory response of the implanted area and histological analysis of internal organs (e.g., heart, liver, spleen, lungs, and kidneys). However, the in vivo fate, distribution, performance, and degradation products of inorganic biomaterials should also be fully considered. Advanced characterization techniques such as high‐throughput assay are expected to evaluate its biosafety from multiple dimensions.

### 5.2 Green and Scale‐Up Synthesis

Currently, inorganic biomaterials are synthesized via hydrothermal, solvothermal sol–gel, mechanical exfoliation and so on; these methods are accompanied with high temperature, high pressure, and multiple reagents.^[^ ^229^ ^]^ On the one hand, the procedure is complex and not suitable for large‐scale synthesis and clinical translation. Hence, it is highly encouraged to develop cheap, safe, and convenient green synthesis methods to synthesize high‐performance inorganic biomaterials with repeatability and reproducibility of physicochemical properties.^[^ ^101^ ^]^ On the other hand, inorganic biomaterials are usually incorporated into organic matrixes such as membranes, hydrogels, and 3D printed scaffolds to exert its biological effects; while, its heterogeneous distribution can affect the physicochemical properties and biological performances.^[^ ^59^ ^]^ Therefore, the optimization of novel functional groups grafting or surface modification strategies still need further exploration.

### 5.3 Exploring the Underlying Mechanism

It must be emphasized that the phenomenon of neural participation and regulated tissue regeneration has just received attention in recent years, and the understanding of related biological mechanisms is still relatively superficial. Biologists typically use pharmaceutical methods such as agonists, antagonists, or genes knockout to study the interaction between innervation and tissue regeneration.^[^ ^157^ ^]^ However, the overall impact on the physiological activity of target tissues is very complex than they observe, which may result in ambiguous outcomes. Besides, the roles of nerves in the different stages of tissue regeneration such as inflammatory, proliferation, and remodeling phase are totally different and need to be systematically studied. Second, after implanting in vivo, the interaction and signals transduction among inorganic biomaterials, innervation, and targeted cells/tissues are still unclear. On the one hand, it is reported that biomaterials regulate tissue regeneration by modulating neural activity instead of directly affecting tissue‐resident cells behaviors. On the other hand, several studies have also found that scaffolds incorporated with neural‐active component could induce the early neural innervation, which exhibited better tissue regeneration and functional recovery outcomes. Hence, it is difficult to accurately analyze the causal relationship between innervation and tissue regeneration. Third, after biomaterials are implanted in vivo, it will cause the immediate response of multiple systems including neural, vascular, and immune system. There is also close interaction among systems such as neuro–immune, neuro–vascular, and immune–vascular, which make it more difficult to analyze the underlying mechanism. To solve this problem, using biomanufacturing technology to create the 3D in vitro pathophysiological model is a practical approach to exploring interactions. Besides, organoids can mimic the composition, 3D structure, and physiological functions of native tissues, which can well recapitulate the pathophysiological process; this may be another promising road. Hence, creating a delicate 3D system containing biomaterials, nerves, and tissues is hopeful to investigate their interactions. Moreover, integrating more advanced characterizations such as machine learning, artificial intelligence, high throughput sequencing, proteomic analysis, gene‐editing, and so on is helpful to understanding the biological mechanisms.

### 5.4 Establishing Evaluation Standards

Successful tissue regeneration involves the participation of many aspects, including tissue‐resident cells differentiation and maturation, reconstruction of vascular network, re‐innervation of nerve fibers, and recovery of physiological functions. Hence, it will be helpful to build a comprehensive standardized protocol to systematically evaluate its in vivo therapeutic efficacy. For example, the dynamic properties of biomaterials in vivo such as mechanical, degradation rates, and swelling profiles have yet to be fully built. Moreover, the functional behaviors including tissue regeneration, vascularization, and re‐innervation activities also need to be carefully established. More importantly, the establishment of standardized protocols should be closely related to animal models including animal species, the size and location of defects, as well as disease status. It is believed that the establishment of systematic standardized protocols can comprehensively evaluate the success of tissue regeneration and provide solid evidence for promoting preclinical/clinical trials.

### 5.5 Embracing New Technologies

It is true that several advanced tools such as machine learning, artificial intelligence (AI), and synthetic biology are expected to revolutionize the development of biomaterials and tissue engineering.^[^ ^230^ ^]^ During the past few decades, biomaterials synthesis has mainly relied on continuous trial and was very time‐consuming and costly. With the development of computational physics methods such as molecular dynamics and density functional theory (DFT), it has become an important tool to direct material synthesis and predict product physicochemical properties. With the accumulation of experimental data and pattern analysis, machine learning can act like an experienced materials scientist for high‐throughput synthesis, analyze results, and make conclusion predictions, even better than humans do, greatly saving time and costs. In addition, scientists have been utilizing artificial intelligence to assist scaffolds preparation, analyze cell–materials interaction, and predict drug screening results, which have greatly increased efficiency and accuracy; while, reducing the time and cost. Recent literature has reviewed the progresses and development of AI‐assisted 3D bioprinting, including bioink preparation, model structure, printing process, and functional control.^[^ ^231^ ^]^ It is expected that AI‐assisted 3D bioprinting may accelerate the clinical translation of 3D bioprinting from bench to bedside. With a growing interest in the concept of programmable biomaterials, synthetic biology has attracted more attention and is a powerful tool to develop new biomaterials with unique physiochemical properties. An example is utilizing the biomineralization process of urease to in situ deposit calcium carbonate on the surface of tissue engineering scaffolds to enhance its osteogenesis activities.^[^ ^232^ ^]^ Moreover, integrating biomaterials and synthetic biology can create “living materials,” which could not only regulate cell behaviors but also modulate by cells in turn.^[^ ^233^ ^]^

### 5.6 Promoting Clinical Translation

The burgeoning of the biomaterials industry has brought unprecedented challenges to the regulators.^[^ ^234^ ^]^ At present, the effects of inorganic‐based biomaterials on innervated tissue regeneration are mainly demonstrated in small animal models such as mice, rats, and rabbits. Hence, it is necessary to conduct large animal experiments, which would not only mimic clinical surgical scenarios but also accurately reflect anatomical, biomechanical, and biological characteristics; thus, maybe providing more substantial evidence for performing preclinical and clinical evaluations. We believe that with the concerted efforts of researchers such as materials scientists, biologists, and doctors, more neural‐active biomaterials will be approved in clinical in the coming years.

In conclusion, innervated tissue regeneration is an emerging and promising research direction, which is expected to restore the physiological functions of damaged tissues. Inorganic‐based material systems have shown great potential in the field of functional tissue regeneration, and their superior performances have been confirmed in several animal models. We expect that this review will help readers understand the current development status and inspire more materials scientists and biologists to participate in this field, collaboratively promoting the development of inorganic biomaterials in both medical research and clinical applications.

## Conflict of Interest

The authors declare no conflict of interest.

## Author Contributions

H.Z. wrote and revised the manuscript and drew the figures; Z.Z. designed and drew the part of figures; and C.W. conceived and supervised the manuscript.
